# High Genetic Diversity and Species Complexity of *Diaporthe* Associated With Grapevine Dieback in China

**DOI:** 10.3389/fmicb.2019.01936

**Published:** 2019-09-02

**Authors:** Ishara S. Manawasinghe, Asha J. Dissanayake, Xinghong Li, Mei Liu, Dhanushka N. Wanasinghe, Jianping Xu, Wensheng Zhao, Wei Zhang, Yueyan Zhou, Kevin D. Hyde, Siraprapa Brooks, Jiye Yan

**Affiliations:** ^1^Beijing Key Laboratory of Environment Friendly Management on Fruit Diseases and Pests in North China, Institute of Plant and Environment Protection, Beijing Academy of Agriculture and Forestry Sciences, Beijing, China; ^2^Center of Excellence in Fungal Research, Mae Fah Luang University, Mueang Chiang Rai, Thailand; ^3^Center for Bioinformatics, School of Life Science and Technology, University of Electronic Science and Technology of China, Chengdu, China; ^4^Key Laboratory for Plant Diversity and Biogeography of East Asia, Kunming Institute of Botany, Chinese Academy of Science, Kunming, China; ^5^Department of Biology, McMaster University, Hamilton, ON, Canada; ^6^College of Plant Protection, China Agricultural University, Beijing, China

**Keywords:** novel species, new host record, network analysis, phylogeography, phomopsis

## Abstract

Grapevine trunk diseases have become one of the main threats to grape production worldwide, with *Diaporthe* species as an emerging group of pathogens in China. At present, relatively little is known about the taxonomy and genetic diversity of Chinese *Diaporthe* populations, including their relationships to other populations worldwide. Here, we conducted an extensive field survey in six provinces in China to identify and characterize *Diaporthe* species in grape vineyards. Ninety-four isolates were identified and analyzed using multi-locus phylogeny. The isolates belonged to eight species, including three novel taxa, *Diaporthe guangxiensis (D. guangxiensis), Diaporthe hubeiensis (D. hubeiensis), Diaporthe viniferae (D. viniferae)*, and three new host records, *Diaporthe gulyae (D. gulyae), Diaporthe pescicola (D. pescicola)*, and *Diaporthe unshiuensis (D. unshiuensis)*. The most commonly isolated species was *Diaporthe eres (D. eres)*. In addition, high genetic diversity was observed for *D. eres* in Chinese vineyards. Haplotype network analysis of *D. eres* isolates from China and Europe showed a close relationship between samples from the two geographical locations and evidence for recombination. In comparative pathogenicity testing, *D. gulyae* was the most aggressive taxon, whereas *D. hubeiensis* was the least aggressive. This study provides new insights into the *Diaporthe* species associated with grapevines in China, and our results can be used to develop effective disease management strategies.

## Introduction

In natural ecosystems, plant pathogens play important roles such as regulating host populations and host plant geographic and ecological distributions. Consequently, they can affect the availability of food sources to other living organisms (Lindahl and Grace, 2015). Most microbial pathogens have short generation times and large population sizes, which can result in high genetic variations and rapid adaptations to environmental stresses and to human-mediated factors such as fungicide resistance (Alberts et al., 2002; Lindahl and Grace, 2015). Hence, it is important to understand the genetic diversity and population variation of plant pathogens to develop sustainable control measures.

Grape is one of the most important fruit crops in China. China is the second largest grape-cultivating country and the top producer in the world (OIV, 2016). In 2016, the total grape cultivation area was estimated at 847 kha, and 14.5 million metric tons of fresh grapes were produced in China (OIV, 2016). Therefore, infectious diseases with significant risks to grape production have drawn broad attention from the grapevine industry. Grapevines are affected by several foliar diseases (Gadoury et al., 2012; Zhang et al., 2017), fruit diseases (Daykin and Milholland, 1984; Hong et al., 2008; Greer et al., 2011; Jayawardena et al., 2015), and trunk diseases (Yan et al., 2013; Dissanayake et al., 2015a,b). Grapevine trunk diseases have drawn considerable attention, as these diseases affect the perennial parts of the vine and can limit grape production for many years (Yan et al., 2013, 2015).

The genus *Diaporthe* Nitschke., belongs to the family *Diaporthaceae*, and is typified by *Diaporthe eres (D. eres)* Nitschke (Senanayake et al., 2017). Following the nomenclature rules Rossman et al. (2014) proposed that the genus name *Diaporthe* over *Phomopsis* as it was introduced first, represents the majority of species. In earlier species names were given to *Diaporthe* taxa based on their host specificity. This resulted in over 100 names listed under the genus *Diaporthe* (http://www.indexfungorum.org/Names/Names.asp and http://www.mycobank.org). With advances in molecular techniques, multi-locus DNA sequence data together with morphological characteristics have been extensively used for the delimitation of *Diaporthe* species (Udayanga et al., 2011; Gomes et al., 2013; Gao et al., 2017). The internal transcribed spacer (ITS), translation elongation factor-1a (EF-1α), β-tubulin, partial histone H3 (HIS), calmodulin (CAL), genes are the most commonly used gene regions for molecular characterization (Udayanga et al., 2011; Gao et al., 2017; Guarnaccia et al., 2018; Yang et al., 2018). Multiple studies have used different gene combinations to resolve the species boundaries in this genus (Udayanga et al., 2011, 2014a,b; Gao et al., 2017; Marin-Felix et al., 2019). Species belonging to genus *Diaporthe* are endophytes, pathogenic, and saprobic on wide range of hosts worldwide (Liu et al., 2015; Hyde et al., 2016; Marin-Felix et al., 2019). They are well-known pathogens on economically important crops (Udayanga et al., 2011). Several common disease among those are dieback on forest trees (Yang et al., 2018), leaf spots on tea (Guarnaccia and Crous, 2017), leaf and pod blights and seed decay on soybean (Udayanga et al., 2015), melanose, stem-end rot, and gummosis on *Citrus* spp. (Mondal et al., 2007; Udayanga et al., 2014a; Guarnaccia and Crous, 2017, 2018) and stem canker on sunflower (Muntañola-Cvetković et al., 1981; Thompson et al., 2011).

Phomopsis cane and leaf spot caused by *Diaporthe* species on grapevine is one of the most complex grapevine trunk diseases worldwide (Úrbez-Torres et al., 2013; Dissanayake et al., 2015a; Guarnaccia et al., 2018). The disease symptoms of Diaporthe Dieback include shoots breaking off at the base, stunting, dieback, loss of vigor, reduced bunch set, and fruit rot (Pine, 1958, 1959; Pscheidt and Pearson, 1989; Pearson and Goheen, 1994; Wilcox et al., 2015). In woods brown to black necrotic irregular-shaped lesions could be observed. Once clusters are infected rachis necrosis and brown, shriveled berries close to harvest could be observed (Pearson and Goheen, 1994). More than one *Diaporthe* species is frequently reported as causative agents from one country (Dissanayake et al., 2015a; Guarnaccia et al., 2018). Currently, 27 species have been identified as causal organisms of Diaporthe dieback in grape-producing countries worldwide (Mostert et al., 2001; Van Niekerk et al., 2005; Udayanga et al., 2011, 2014a,b; White et al., 2011; Baumgartner et al., 2013; Úrbez-Torres et al., 2013; Hyde et al., 2014; Dissanayake et al., 2015a; Guarnaccia et al., 2018; Lesuthu et al., 2019). Even though these species characterized under the one disease, disease symptoms, and aggressiveness are varying according to the species. *Diaporthe ampelina* (*D. ampelina)* has a long history as the most common and severe pathogenic species together with *D. amygdali* (Mostert et al., 2001; Van Niekerk et al., 2005). *Diaporthe ampelina* and *Diaporthe kyushuensis (D. kyushuensis)* are the causal agent of grapevine swelling arm (Kajitani and Kanematsu, 2000; Van Niekerk et al., 2005). *Diaporthe perjuncta (D. perjuncta)* and *D. ampelina* caused cane bleaching (Kuo and Leu, 1998; Kajitani and Kanematsu, 2000; Mostert et al., 2001; Van Niekerk et al., 2005; Rawnsley et al., 2006). Lesuthu et al. (2019) showed that *D. ampelina, Diaporthe novem (D. novem)*, and *Diaporthe nebulae (D. nebulae)* as the most virulent species of *Diaporthe* associated with grapevines in South Africa. *Diaporthe eres* was found as a weak to moderate pathogen in several different studies (Kaliterna et al., 2012; Baumgartner et al., 2013). These results indicate the complexity and high species richness of *Diaporthe* associated with the grapevines. Up to now in China four *Diaporthe* species have been reported causing grapevine dieback (Dissanayake et al., 2015a). Those are *D. eres, Diaporthe hongkongensis (D. hongkongensis), Diaporthe phaseolorum (D. phaseolorum)*, and *Diaporthe sojae (D. sojae)*. Their taxonomic placements and pathogenicity under a controlled environment were also studied.

The study conducted by Guarnaccia et al. (2018) showed that species of *Diaporthe* also associated as endophytes on grapes as well. In that study they observed that *Diaporthe bohemiae (D. bohemiae)*, which was isolated from grape was unable to induce lesions. In addition to grapevines, *Diaporthe* have been reported on broad range of hosts (Udayanga et al., 2011). However, the most important charter is the ability of endophytic *Diaporthe* species to be opportunistic pathogens. Huang et al. (2015) observed that some *Diaporthe* species associated with citrus in China shown to act as opportunistic plant pathogens. *Diaporthe foeniculina (D. foeniculina)* has been found as both endophyte and opportunistic pathogen on various herbaceous weeds, ornamentals, and fruit trees (Udayanga et al., 2014a; Guarnaccia et al., 2016). So far it is not confirmed the factor that driven into pathogenicity from endophytes either due to environmental changes or the reduction of host's defense. Therefore, further studies are required to understand this in both field level and genomic level.

However, the genetic diversity of *Diaporthe* spp. associated with *Vitis* spp., relationships among isolates from different geographical regions, and relationships among isolates from China and those from other countries were not investigated. Therefore, to expand our knowledge on these issues, we performed an extensive field survey to isolate and identify *Diaporthe* species associated with grapevine dieback in China. We reconstructed a phylogenetic tree for the genus *Diaporthe*. The present study analyzed the genetic diversity of *Diaporthe* species associated with grapevines in China and constructed haplotype networks for *Diaporthe* species from different geographical origins for the first time. Finally, we analyzed the relationship between *Diaporthe* species from European and Chinese grape vineyards, as Diaporthe dieback is becoming an emerging trunk disease in both regions (Guarnaccia et al., 2018).

## Materials and Methods

### Sampling and Pathogen Isolation

**Figure 1:**
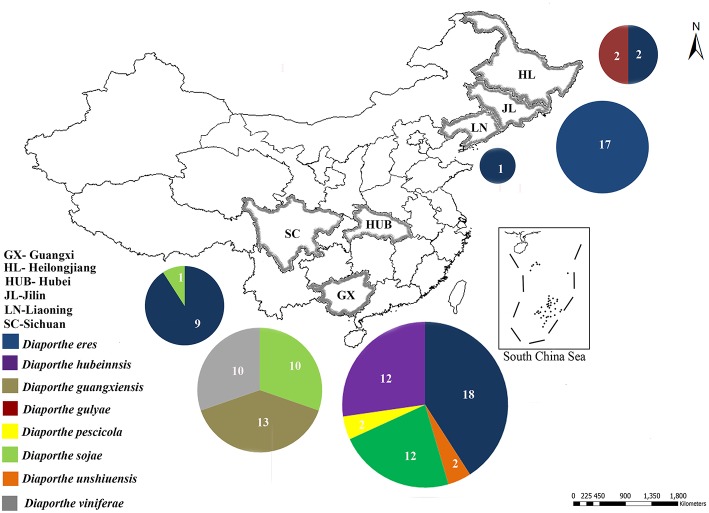
Sample collection sites of Diaporthe dieback in six provinces in China. Circles represent the association frequency of each species in each population sampled, and the number of isolates analyzed in each population is given inside the respective slice.

**Figure 2:**
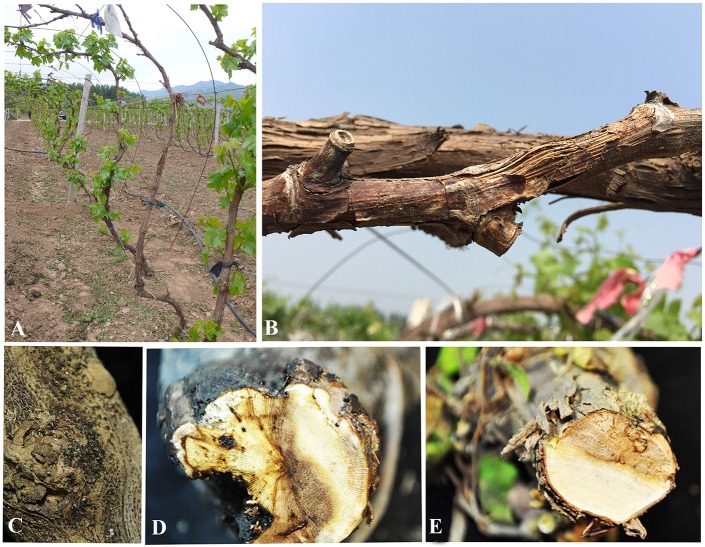
Symptoms of Diaporthe dieback. **(A,B)** Field symptoms on trunks and shoots, **(C)** appearance of fruiting bodies on trunk surface, and **(D,E)** cross sections of infected trunks.

Field surveys were conducted during 2014 and 2015 in 20 vineyards in the six following provinces in China: Guangxi, Heilongjiang, Hubei, Jilin, Liaoning, and Sichuan (Figure 1). Samples were collected from symptomatic grapevine woody branches that exhibited bark discoloration, shoots breaking off at the base, stunting, wedge-shaped cankers, and light brown streaking of the wood from the following *Vitis vinifera* (*V. vinifera)* cultivars: Centennial Seedless, Red Globe, and Summer Black (Figure 2). Symptomatic tissue samples were collected into zip-lock plastic bags that contained wet sterilized tissue papers to maintain humidity. Once the samples were taken into the laboratory, infected trunks or shoots were photographed, and symptoms, location, and other relevant data were documented. The fungal pathogens were isolated using the following procedures. Infected shoots/trunks were cut into small pieces (1–3 mm thick). These pieces were then surface-sterilized by dipping into 70% ethanol for 30 s and then transferred into 1% NaOCl for 1 min. This step was followed by two washes with sterile distilled water. Once the wood pieces were dried, they were placed onto potato dextrose agar (PDA) plates supplemented with ampicillin (0.1 g L^−1^) and incubated at 25°C. After 5–7 days of incubation, hyphal tips of fungi immerging from wood pieces were transferred onto new PDA plates and incubated until they produce conidia. Once the conidia were developed single spore isolation was done. For the strains do not developed conidia after 4 weeks two-three times hyphal tip isolation was done. All the pure cultures obtained in this study were deposited in the culture collection of Institute of Plant and Environment Protection of Beijing Academy of Agriculture and Forestry Sciences (JZB culture collection) at 4°C.

### DNA Extraction, PCR Amplification, and Sequence Assembly

Approximately 10 mg of aerial mycelium was scraped from 5–7 days old isolates grown on PDA (Potato Dextrose Agar) at 25°C. Total genomic DNA was extracted using the DNeasy Plant Mini Kit (QIAGEN GmbH, QIAGEN Strasse 1, 40742 Hilden, Germany). For species confirmation, the internal transcribed spacer (ITS) regions were sequenced for all isolates. The obtained sequences were compared to those in GenBank using the MegaBLAST tool (https://blast.ncbi.nlm.nih.gov/Blast.cgi). After isolates were confirmed as belonging to the genus *Diaporthe*, six additional gene regions, those encoding translation elongation factor-1α (EF-1α), β-tubulin, calmodulin (CAL), partial histone H3 (HIS), partial actin (ACT), and DNA-lyase (Apn2), were sequenced. Table 1 presents the primer pairs with their respective amplification conditions for each of the above gene regions. PCR mixtures of 25 μl total volume consisted of 0.3 μl of TaKaRa Ex-Taq DNA polymerase, 2.5 μl of 10 × Ex-Taq DNA polymerase buffer, 3.0 μl of dNTPs, 2 μl of genomic DNA, 1 μl of each primer, and 15.2 ddH2O. The PCRs were conducted in a Bio-Rad C1000 thermal cycler (Germany). The resulting products were visualized on a 1% agarose gel stained with ethidium bromide under UV light using a Gel DocTM XR Molecular Imager (Bio Rad, USA). All positive amplicons were sequenced by Beijing Biomed Gene Technology Co LTD. The sequence quality was confirmed by checking chromatograms using BioEdit v. 5 (Hall, 2006). Sequences were obtained using both forward and reverse primers, and consensus sequences were generated using DNAStar v. 5.1 (DNASTAR, Inc.). The sequence data generated in the present study have been deposited in GenBank (Table 2).

**Table 1:** Gene regions and respective primer pairs used in the study.

**Gene region**	**Primers**	**Sequence 5′-3′**	**Optimized PCR protocols**	**References**
ACT	ACT-512F	ATGTGCAAGGCCGGTTTCGC	95°C: 5 min, (95°C: 30 s, 55°C: 50 s,72°C: 1 min) × 39 cycles 72°C: 10 min	Carbone and Kohn, 1999
	ACT-783R	TACGAGTCCTTCTGGCCCAT		
Apn2 (DNA lyase	apn2fw2	GCMATGTTYGAMATYCTGGAG	94°C: 1 min, (95°C: 30 s, 54°C: 50 s, 72°C: 1 min) × 39 cycles 72°C: 10 min	Udayanga et al., 2012a,b
	apn2rw2	CTT GGTCTCCCAGCAGGTG AAC		
CAL	CAL-228F	GAGTTCAAGGAGGCCTTCTCCC	95°C: 5 min, (95°C: 30 s, 55°C: 50 s, 72°C: 1 min) × 34 cycles 72°C: 10 min	Carbone and Kohn, 1999
	CAL-737R	CATCTTCTGGCCATCATGG		
EF1-α	EF1-728F	CATCGAGAAGTTCGAGAAGG	95°C: 5 min, (95°C: 30 s, 58°C: 30 s, 72°C: 1 min) × 34 cycles 72°C: 10 min	Carbone and Kohn, 1999
	EF1-986R	TACTTGAAGGAACCCTTACC		Udayanga et al., 2012a,b
HIS	CYLH3F	AGGTCC ACTGGTGGCAAG	96°C: 5 min, (96°C: 30 s, 58°C: 50 s, 72°C: 1 min) × 30 cycles 72°C: 5 min	Crous et al., 2004
	H3-1b	GCGGGCGAGCTGGATGTCCTT		Glass and Donaldson, 1995
ITS	ITS1	TCCGTAGGTGAACCTGCGG	94°C: 5 min, (94°C: 30 s, 55°C: 50 s, 72°C: 1 min) × 34 cycles 72°C: 10 min	White et al., 1990
	ITS4	TCCTCCGCTTATTGATATGC		Udayanga et al., 2012a,b
β-tubulin	BT2a	GGTAACCAAATCGGTGCTGCTTTC	94°C: 5 min, (94°C: 30 s, 58°C: 50 s, 72°C: 1 min) × 34 cycles 72°C: 10 min	Glass and Donaldson, 1995
	Bt2b	ACCCTCAGTGTAGTGACCCTTGGC		Udayanga et al., 2012a,b

**Table 2:** *Diaporthe* species isolated and characterized in the present study.

**No**	**Species**	**Location**	**Year**	**JZB number**	**Sequence data**			
					**ITS**	**β-tubulin**	**CAL**	**EF-1α**
01	*Diaporthe eres*	Sichuan	2015	JZB320020^*^	–	MK500169	MK500062	MK523586
02		Sichuan	2015	JZB320021^*^	MK335710	MK500170	MK500063	MK523587
03		Sichuan	2015	JZB320022^*^	MK335711	MK500171	MK500064	MK523588
04		Sichuan	2015	JZB320023^*^	MK335712	MK500172	MK500065	MK523589
05		Sichuan	2015	JZB320024^*^	MK335713	MK500173	MK500066	–
06		Sichuan	2015	JZB320026	MK335714	MK500174	MK500067	MK523591
07		Sichuan	2015	JZB320027^*^	MK335715	MK500175	MK500068	MK523619
08		Sichuan	2015	JZB320028^*^	MK335716	MK500176	MK500069	MK523592
09		Sichuan	2015	JZB320029^*^	MK335717	MK500177	MK500070	MK523620
10		Lioning	2015	JZB320030	MK335718	MK500178	MK500071	MK523621
11		Hubei	2015	JZB320033^*^	MK335719	MK500179	MK500072	MK523622
12		Hubei	2015	JZB320034^*^	MK335720	MK500180	MK500073	MK523623
13		Hubei	2015	JZB320035^*^	MK335721	MK500181	MK500074	MK523593
14		Hubei	2015	JZB320036^*^	MK335722	MK500182	MK500075	–
15		Hubei	2015	JZB320037^*^	MK335723	MK500183	MK500076	–
16		Hubei	2015	JZB320038^*^	MK335724	MK500184	MK500077	MK523594
17		Hubei	2015	JZB320039^*^	MK335725	MK500185	MK500078	MK523595
18		Hubei	2015	JZB320040^*^	MK335726	MK500186	MK500079	MK523596
19		Hubei	2015	JZB320041^*^	MK335727	MK500187	MK500080	–
20		Hubei	2015	JZB320043^*^	MK335728	MK500188	MK500081	MK523624
21		Hubei	2015	JZB320044^*^	MK335729	MK500189	MK500082	–
22		Hubei	2015	JZB320045^*^	MK335730	–	MK500083	MK523597
23		Hubei	2015	JZB320046^*^	MK335731	MK500190	MK500084	MK523598
24		Hubei	2015	JZB320047	MK335732	MK500191	MK500085	–
25		Hubei	2015	JZB320048^*^	MK335733	MK500192	MK500086	MK523599
26		Hubei	2015	JZB320049^*^	MK335734	MK500193	MK500087	MK523625
27		Hubei	2015	JZB320051^*^	MK335735	MK500194	MK500088	MK523600
28		Hubei	2015	JZB320052	MK335736	MK500195	MK500089	–
29		Heilongjiang	2015	JZB320053^*^	MK335737	MK500196	MK500090	MK523601
30		Jilin	2015	JZB320054	MK335738	MK500197	MK500091	MK523602
31		Jilin	2015	JZB320055^*^	MK335739	MK500198	MK500092	MK523617
32		Jilin	2015	JZB320056^*^	MK335740	MK500199	MK500093	MK523618
33		Jilin	2015	JZB320057^*^	MK335741	MK500200	MK500094	MK523603
34		Jilin	2015	JZB320058^*^	MK335742	MK500201	MK500095	MK523604
35		Jilin	2015	JZB320059^*^	MK335743	MK500202	MK500096	MK523605
36		Jilin	2015	JZB320060	MK335744	MK500203	MK500097	MK523606
37		Jilin	2015	JZB320061^*^	MK335745	MK500204	MK500098	MK523607
38		Jilin	2015	JZB320062^*^	MK335746	MK500205	MK500099	MK523614
39		Jilin	2015	JZB320063^*^	MK335747	MK500206	MK500100	MK523608
40		Jilin	2015	JZB320064^*^	MK335748	MK500207	MK500101	MK523609
41		Jilin	2015	JZB320065	MK335749	MK500208	MK500102	MK523615
42		Jilin	2015	JZB320066	MK335750	MK500209	MK500103	MK523610
43		Jilin	2015	JZB320067	MK335751	MK500210	MK500104	MK523611
44		Jilin	2015	JZB320068^*^	MK335752	MK500211	MK500105	MK523612
45		Jilin	2015	JZB320069^*^	MK335753	MK500212	MK500106	MK523616
46		Jilin	2015	JZB320070^*^	MK335754	MK500213	–	MK523613
47	*Diaporthe guangxiensis*	Guangxi	2015	JZB320082	MK335760	MK500156	MK736715	MK523557
48		Guangxi	2015	JZB320083	MK335761	MK500157	MK736716	MK523558
49		Guangxi	2015	JZB320084	MK335762	MK500158	MK736717	–
50		Guangxi	2015	JZB320085	MK335763	MK500159	MK736718	–
51		Guangxi	2015	*JZB320086*	MK335764	MK500160	MK736719	MK523559
52		Guangxi	2015	JZB320087^*^	MK335765	MK500161	MK736720	MK523560
53		Guangxi	2015	JZB320088	MK335766	MK500162	MK736721	MK523561
54		Guangxi	2015	JZB320089	MK335767	MK500163	MK736722	MK523562
55		Guangxi	2015	JZB320090	MK335768	MK500164	MK736723	MK523563
56		Guangxi	2015	JZB320091^*^	MK335769	MK500165	MK736724	MK523564
57		Guangxi	2015	JZB320092	MK335770	MK500166	MK736725	–
58		Guangxi	2015	JZB320093^*^	MK335771	MK500167	MK736726	MK523565
59		Guangxi	2015	JZB320094^*^	**MK335772**	**MK500168**	**MK736727**	**MK523566**
60	*Diaporthe gulyae*	Heilongjiang	2015	*JZB320118*	KY400792	KY400856	–	KY400824
61		Heilongjiang	2015	*JZB320119*	KY400793	KY400857	–	KY400825
62	*Diaporthe hubeiensis*	Hubei	2015	JZB320120	MK335806	MK500144	MK500232	MK523567
63		Hubei	2015	JZB320121^*^	MK335807	MK500146	MK500233	MK523568
64		Hubei	2015	JZB320122^*^	MK335808	MK500147	MK500234	MK523569
65		Hubei	2015	JZB320123^*^	**MK335809**	**MK500148**	**MK500235**	**MK523570**
66		Hubei	2015	JZB320124^*^	MK335810	MK500149	MK500236	MK523571
67		Hubei	2015	JZB320125^*^	MK335811	MK500150	MK500237	–
68		Hubei	2015	JZB320126	MK335812	MK500151	MK500238	–
69		Hubei	2015	JZB320127^*^	MK335813	MK500152	MK500239	MK523572
70		Hubei	2015	JZB320128^*^	MK335814	MK500153	MK500240	MK523573
71		Hubei	2015	JZB320139^*^	MK335815	MK500154	MK500241	–
72		Hubei	2015	*JZB320130*	MK335816	MK500155	MK500242	–
73	*Diaporthe pescicola*	Hubei	2015	*JZB320095*	KY400784	KY400890	–	KY400817
74		Hubei	2015	*JZB320096*	KY400785	KY400891	–	KY400831
75	*Diaporthe sojae*	Sichuan	2015	JZB320097	MK335826	MK500126	MK500214	MK523574
76		Hubei	2015	*JZB320098*	MK335827	MK500127	MK500215	MK523575
77		Hubei	2015	JZB320099	MK335828	MK500128	MK500216	MK523576
78		Hubei	2015	JZB320100	MK335829	–	MK500217	–
79		Guangxi	2015	JZB320101	MK335830	MK500129	MK500218	MK523577
80		Guangxi	2015	JZB320102	MK335831	MK500130	MK500219	MK523578
81		Guangxi	2015	JZB320103	MK335832	MK500131	MK500220	MK523579
82		Guangxi	2015	JZB320104	MK335833	MK500132	MK500221	MK523580
83		Guangxi	2015	JZB320105	MK335834	MK500133	MK500222	–
84		Guangxi	2015	JZB320106	MK335835	MK500134	MK500223	–
85		Guangxi	2015	JZB320107	MK335836	MK500135	MK500224	–
86		Guangxi	2015	*JZB320108*	MK335837	MK500136	MK500225	MK523581
87		Guangxi	2015	JZB320109	MK335838	MK500137	MK500226	MK523582
88		Guangxi	2015	JZB320110	MK335839	MK500138	MK500227	–
89		Hubei	2015	JZB320111	MK335840	MK500139	MK500228	–
90		Hubei	2015	JZB320112	MK335841	MK500140	MK500228	MK523583
91		Hubei	2015	JZB320113	MK335842	MK500141	MK500230	MK523584
92		Hubei	2015	JZB320114	MK335843	MK500142	MK500231	MK523585
93		Hubei	2015	JZB320115	**–**	MK500143	–	–
94	*Diaporthe unshiuensis*	Hubei	2015	*JZB320116*	KY400790	KY400854	–	KY400822
95		Hubei	2015	*JZB320117*	KY400791	KY400855	–	KY400823
96	*Diaporthe viniferae*	Guangxi	2015	JZB320071^*^	**MK341551**	**MK500112**	**MK500119**	**MK500107**
97		Guangxi	2015	*JZB320072*	MK341552	MK500113	MK500120	MK500108
98		Guangxi	2015	JZB320076^*^	MK341553	MK500115	MK500122	–
99		Guangxi	2015	JZB320077	MK341554	MK500116	MK500123	MK500109
100		Guangxi	2015	JZB320078^*^	MK341555	MK500117	MK500124	MK500110
101		Guangxi	2015	JZB320079^*^	MK341556	MK500118	MK500125	MK500111

*JZB: Culture collection of Institute of Plant and Environment Protection, Beijing Academy of Agriculture and Forestry Sciences, Beijing 100097, China. Ex-type cultures are indicated in bold. Isolates used in pathogenicity test are Italic. ITS, internal transcribed spacers 1 and 2 together with 5.8S nrDNA; β-tubulin, partial beta-tubulin gene; CAL, partial calmodulin gene; EF-1α, partial translation elongation factor 1-α gene*.

**Figure 3:**
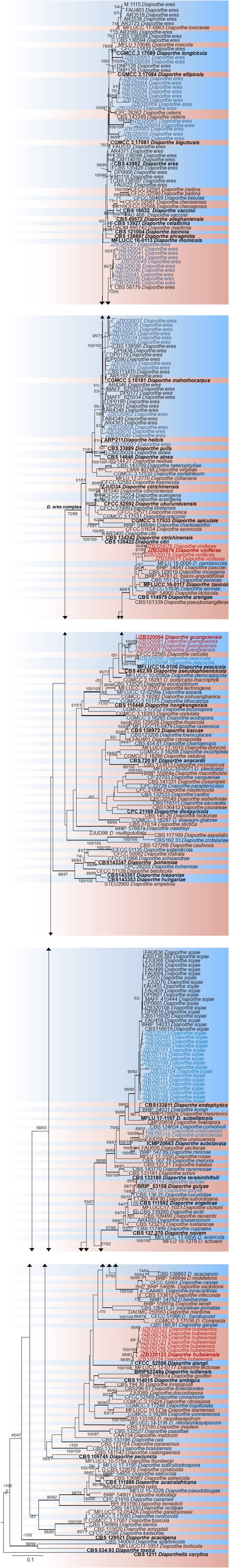
RAxML tree based on analysis of a combined dataset of ITS, β-tubulin, CAL, and EF-1α sequences. Bootstrap support values for ML and MP equal to or >50% are shown as ML/MP above the nodes. The isolates obtained for the present study are shown in blue for already known species, and novel taxa are shown in red. Ex-type strains are indicated in bold. The tree is rooted using *Diaporthella corylina*. The scale bar represents the expected number of nucleotide substitutions per site.

* *Strains used in phylogenetic analysis (Figure 3)*.

### Phylogenetic Analyses

For the phylogenetic analyses, reference sequences representing related taxa in *Diaporthe* were downloaded from GenBank (Guarnaccia et al., 2018; Yang et al., 2018; Table 3) and aligned with the sequences obtained in this study (Table 2). The sequences were aligned using MAFFT (Katoh and Toh, 2010) (http://www.ebi.ac.uk/Tools/msa/mafft/) and manually adjusted using BioEdit v. 5 (Hall, 2006) whenever necessary. Phylogenetic relationships were inferred using maximum parsimony (MP) implemented in PAUP (v4.0) (Swofford, 2003), maximum likelihood (ML) in RAxML (Silvestro and Michalak, 2010) and Bayesian analyses in MrBayes v. 3.0b4 (Ronquist and Huelsenbeck, 2003). In phylogenetic analysis, single-gene trees were constructed first using ML in RAxML. The phylogenetic tree topologies for different gene fragments were compared for evidence of incongruences with a focus on comparing branches with high bootstrap values. If no conflict was observed, a combined phylogenetic tree was generated.

**Table 3:** *Diaporthe* taxa used in the phylogenetic analysis.

**Species**	**Isolate**	**Host**	**Location**	**GenBank accession numbers**			
				**ITS**	**β-tubulin**	**CAL**	**EF-1α**
*D. acaciarum*	CBS 138862	*Acacia tortilis*	Tanzania	KP004460	KP004509	N/A	N/A
*D. acaciigena*	**CBS 129521**	*Acacia retinodes*	Australia	KC343005	KC343973	KC343247	KC343731
*D. acericola*	MFLUCC 17-0956	*Acer negundo*	Italy	KY964224	KY964074	KY964137	KY964180
*D. acerigena*	CFCC 52554	*Acer tataricum*	China	MH121489	N/A	MH121413	MH121531
	CFCC 52555	*Acer tataricum*	China	MH121490	N/A	MH121414	MH121532
*D. acutispora*	CGMCC 3.18285	*Coff* sp.	China	KX986764	KX999195	KX999274	KX999155
*D. alangii*	**CFCC 52556**	***Alangium kurzii***	**China**	**MH121491**	**MH121573**	**MH121415**	**MH121533**
*D. alleghaniensis*	**CBS 495.72**	***Betula alleghaniensis***	**Canada**	**KC343007**	**KC343975**	**KC343249**	**KC343733**
*D. alnea*	**CBS 146.46**	***Alnus*** **sp**.	**Netherlands**	**KC343008**	**KC343976**	**KC343250**	**KC343734**
*D. ambigua*	**CBS 114015**	***Pyrus communis***	**South Africa**	**KC343010**	**KC343978**	**KC343252**	**KC343736**
*D. ampelina*	STEU2660	*Vitis vinifera*	France	AF230751	JX275452	AY745026	AY745056
*D. amygdali*	**CBS 115620**	***Prunus persica***.	**USA**	**KC343020**	**KC343988**	**KC343262**	**KC343746**
	CBS111811	*Vitis vinifera*	South Africa	KC343019	KC343987	KC343261	KC343745
	CBS120840	*Prunus salicina*	South Africa	KC343021	KC343989	KC343263	KC343747
	CBS 126679	*Prunus dulcis*	Portugal	KC343022	KC343990	KC343264	KC343748
*D. anacardii*	**CBS 720.97**	***Anacardium occidentale***	**East Africa**	**KC343024**	**KC343992**	**KC343266**	**KC343750**
*D. angelicae*	**CBS 111592**	***Heracleum sphondylium***	**Austria**	**KC343027**	**KC343995**	**KC343269**	**KC343753**
*D. apiculate*	**CGMCC 3 17533**	***Camellia sinensis***	**China**	**KP267896**	**KP293476**	**N/A**	**KP267970**
	LC3187	*Camellia sinensis*	China	KP267866	KP293446	N/A	KP267940
*D. arengae*	**CBS 114979**	***Arenga engleri***	**Hong Kong**	**KC343034**	**KC344002**	**KC343276**	**KC343760**
*D. aquatica*	IFRDCC 3051	*Aquatic habitat*	China	JQ797437	N/A	N/A	N/A
*D. arctii*	CBS 139280	*Arctium lappa*	Austria	KJ590736	KJ610891	KJ612133	KJ590776
*D. arengae*	CBS 114979	*Arenga enngleri*	Hong Kong	KC343034	KC344002	KC343276	KC343760
*D. aseana*	MFLUCC 12-0299a	*Unknown dead leaf*	Thailand	KT459414	KT459432	KT459464	KT459448
*D. asheicola*	CBS 136967	*Vaccinium ashei*	Chile	KJ160562	KJ160518	KJ160542	KJ160594
*D. aspalathi*	CBS 117169	*Aspalathus linearis*	South Africa	KC343036	KC344004	KC343278	KC343762
*D. australafricana*	**CBS 111886**	***Vitis vinifera***	**Australia**	**KC343038**	**KC344006**	**KC343280**	**KC343764**
*D. baccae*	**CBS 136972**	***Vaccinium*** **sp**.	**Italy**	**KJ160565**	**N/A**	**N/A**	**KJ160597**
*D. batatas*	CBS 122.21	*Ipomoea batatas*	USA	KC343040	KC344008	KC343282	KC343766
*D. beilharziae*	BRIP 54792	*Indigofera australis*	Australia	JX862529	KF170921	N/A	JX862535
*D. benedicti*	BPI 893190	*Salix* sp.	USA	KM669929	N/A	KM669862	KM669785
*D. betulae*	CFCC 50469	*Betula platyphylla*	China	KT732950	KT733020	KT732997	KT733016
*D. betulicola*	CFCC 51128	*Betula albo-sinensis*	China	KX024653	KX024657	KX024659	KX024655
	CFCC 52560	*Betula albo- sinensis*	China	MH121495	MH121577	MH121419	MH121537
*D. betulina*	CFCC 52561	*Betula costata*	China	MH121496	MH121578	MH121420	MH121538
*D. bicincta*	**CBS 121004**	***Juglans*** **sp**.	**USA**	**KC343134**	**KC344102**	**KC343376**	**KC343860**
*D. biconispora*	CGMCC 3.17252	*Citrus grandis*	China	KJ490597	KJ490418	KJ490539	KJ490476
*D. biguttulata*	CFCC 52584	*Juglans regia*	China	MH121519	MH121598	MH121437	MH121561
*D. biguttusis*	**CGMCC 317081**	***Lithocarpus glabra***	**China**	**KF576282**	**KF576306**	**N/A**	**KF576257**
	CGMCC 317081	*Lithocarpus glabra*	China	KF576283	KF576307	N/A	KF576258
*D. bohemiae*	**CBS 1433477**	***Vitis vinifera***	**Czech Republic**	**MG281015**	**MG281188**	**MG281710**	**MG281536**
	CBS 1433478	*Vitis vinifera*	Czech Republic	MG281016	MG281189	MG281711	MG281537
*D. brasiliensis*	CBS 133183	*Aspidosperma* sp.	Brazil	KC343042	KC344010	KC343284	KC343768
*D. caatingaensis*	CBS 141542	*Tacinga inamoena*	Brazil	KY085927	KY115600	N/A	KY115603
*D. camptothecicola*	CFCC 51632	*Camptotheca* sp.	China	KY203726	KY228893	KY228877	KY228887
*D. canthii*	CBS 132533	*Canthium inerme*	South Africa	JX069864	KC843230	KC843174	KC843120
*D. caryae*	CFCC 52563	*Carya illinoensis*	China	MH121498	MH121580	MH121422	MH121540
	CFCC 52564	*Carya illinoensis*	China	MH121499	MH121581	MH121423	MH121541
*D. cassines*	CPC 21916	*Cassine peragua*	South Africa	KF777155	N/A	N/A	KF777244
*D. caulivora*	CBS 127268	*Glycine max*	Croatia	KC343045	KC344013	KC343287	KC343771
*D. celeris*	CBS143349	***Vitis vinifera***	**Czech Republic**	**MG281017**	**MG281190**	**MG281712**	**MG281538**
	CBS143350	*Vitis vinifera*	Czech Republic	MG281018	MG281191	MG281713	MG281539
*D. celastrina*	**CBS 139.27**	***Celastrus*** **sp**.	**USA**	**KC343047**	**KC344015**	**KC343289**	**KC343773**
*D. cf nobilis*	CBS 113470	*Castanea sativa*	South Korea	KC343146	KC344114	KC343388	KC343872
	CBS 587 79	*Pinus pantepella*	Japan	KC343153	KC344121	KC343395	KC343879
*D. cercidis*	CFCC 52565	*Cercis chinensis*	China	MH121500	MH121582	MH121424	MH121542
*D. chamaeropis*	CBS 454.81	*Chamaerops humilis*	Greece	KC343048	KC344016	KC343290	KC343774
*D. charlesworthii*	BRIP 54884m	*Rapistrum rugostrum*	Australia	KJ197288	KJ197268	N/A	KJ197250
*D. chensiensis*	CFCC 52567	*Abies chensiensis*	China	MH121502	MH121584	MH121426	MH121544
	CFCC 52568	*Abies chensiensis*	China	MH121503	MH121585	MH121427	MH121545
*D. cichorii*	MFLUCC 17-1023	*Cichorium intybus*	Italy	KY964220	KY964104	KY964133	KY964176
*D. cinnamomi*	CFCC 52569	*Cinnamomum* sp.	China	MH121504	MH121586	N/A	MH121546
*D. cissampeli*	CBS 141331	*Cissampelos capensis*	South Africa	KX228273	KX228384	N/A	N/A
*D. citri*	**CBS 135422**	***Citrus*** **sp**.	**Florida, USA**	**KC843311**	**KC843187**	**KC843157**	**KC843071**
	AR4469	*Citrus* sp.	Florida, USA	KC843321	KC843167	KC843197	KC843081
*D. citriasiana*	CGMCC 3.15224	*Citrus unshiu*	China	JQ954645	KC357459	KC357491	JQ954663
*D. citrichinensis*	**ZJUD34**	***Citrus*** **sp**.	**China**	**JQ954648**	**N/A**	**KC357494**	**JQ954666**
	ZJUD85	*Citrus* sp.	China	KJ490620	KJ490441	N/A	KJ490499
*D. collariana*	MFLU 17-2770	*Magnolia champaca*	Thailand	MG806115	MG783041	MG783042	MG783040
*D. compacta*	CGMCC 3.17536	*Camellia sinensis*	China	KP267854	KP293434	N/A	KP267928
*D. conica*	CFCC 52571	*Alangium chinense*	China	MH121506	MH121588	MH121428	MH121548
*D. convolvuli*	CBS 124654	*Convolvulus arvensis*	Turkey	KC343054	KC344022	KC343296	KC343780
*D. crotalariae*	CBS 162.33	*Crotalaria spectabilis*	USA	KC343056	KC344024	KC343298	KC343782
*D. cucurbitae*	CBS 136.25	*Arctium* sp.	Unknown	KC343031	KC343999	KC343273	KC343757
*D. cuppatea*	CBS 117499	*Aspalathus linearis*	South Africa	KC343057	KC344025	KC343299	KC343783
*D. cynaroidis*	CBS 122676	*Protea cynaroides*	South Africa	KC343058	KC344026	KC343300	KC343784
*D. cytosporella*	FAU461	*Citrus limon*	Italy	KC843307	KC843221	KC843141	KC843116
*D. diospyricola*	**CPC 21169**	***Diospyros whyteana***	**South Africa**	**KF777156**	**N/A**	**N/A**	**N/A**
*D. discoidispora*	ZJUD89	*Citrus unshiu*	China	KJ490624	KJ490445	N/A	KJ490503
*D. dorycnii*	MFLUCC 17-1015	*Dorycnium hirsutum*	Italy	KY964215	KY964099	N/A	KY964171
*D. elaeagni-glabrae*	CGMCC 3.18287	*Elaeagnus glabra*	China	KX986779	KX999212	KX999281	KX999171
*D.ellipicola*	**CGMC 3 17084**	***Lithocarpus glabra***	**China**	**KF576270**	**KF576291**	**N/A**	**KF576245**
*D.endophytica*	**CBS133811**	***Schinus terebinthifolius***	**Brazil**	**KC343065**	**KC343065**	**KC343307**	**KC343791**
	LGMF911	*Schinus terebinthifolius*	Brazil	KC343066	KC344034	KC343308	KC343792
*D.eres*	AR3519	*Corylus avellana*	Austria	KJ210523	KJ420789	KJ435008	KJ210547
	CBS 109767 = AR3538	*Acer* sp.	Austria	DQ491514 KC344043		KC343317 KC343801	
	AR3560	*Viburnum* sp.	Austria	JQ807425	KJ420795	KJ435011	JQ807351
	AR3723	*Rubus fruticosus*	Austri	JQ807428 KJ420793		KJ435024 JQ807354	
	AR4346	*Prunus mume*	Korea	JQ807429	KJ420823	KJ435003	JQ807355
	AR4373	*Ziziphus jujuba*	Korea	JQ807442	KJ420798	KJ435013	JQ807368
	AR4348	*Prunus persica*	Korea	JQ807431	KJ420811	KJ435004	JQ807357
	AR4363	*Malus* sp.	Korea	JQ807436	KJ420809	KJ435033	JQ807362
	AR4369	*Pyrus pyrifolia*	Korea	JQ807440	KJ420813	KJ435005	JQ807366
	AR4371	*Malus pumila*	Korea	JQ807441	KJ420796	KJ435034	JQ807367
	**AR5193**	***Ulmus*** **sp**.	**Germany**	**KJ210529**	**KJ420799**	**KJ434999**	**KJ210550**
	AR5197	*Rhododendron* sp.	Germany	KJ210531	KJ420812	KJ435014	KJ210552
	CBS113470	*Castanea sativa*	Australia	KC343146	KC344114	KC343388	KC343872
	CBS135428	*Juglans cinerea*	USA	KC843328	KC843229	KC843155	KC843121
	CBS138594	*Ulmus laevis*	Germany	KJ210529	KJ420799	KJ434999	KJ210550
	CBS138595	*Ulmus laevis*	Germany	KJ210533	KJ420817	KJ435006	KJ210554
	CBS138597	*Vitis vinifera*	France	KJ210518	KJ420783	KJ434996	KJ210542
	CBS138598	*Ulmus* sp.	USA	KJ210521	KJ420787	KJ435027	KJ210545
	CBS138599	*Acer nugundo*	Germany	KJ210528	KJ420830	KJ435000	KJ210549
	CBS439.82	*Cotoneaster* sp.	UK	FJ889450	JX275437	JX197429	GQ250341
	DNP128.1	*Castaneae mollissimae*	China	JF957786	KJ420801	KJ435040	KJ210561
	DNP129	*Castanea mollissima*	China	JQ619886	KJ420800	KJ435039	KJ210560
	DP0177	*Pyrus pyrifolia*	New Zealand	JQ807450	KJ420820	KJ435041	JQ807381
	DP0179	*Pyrus pyrifolia*	New Zealand	JQ807452	KJ420803	KJ43502	JQ807383
	DP0180	*Pyrus pyrifolia*	New Zealand	JQ807453	KJ420804	KJ435029	JQ807384
	DP0438	*Ulmus minor*	Austria	KJ210532	KJ420816	KJ435016	KJ210553
	FAU506	*Cornus florida*	USA	KJ210526	KJ420792	KJ435012	JQ807403
	DP0590	*Pyrus pyrifolia*	New Zealand	JQ807464	KJ420810	KJ435037	JQ807394
	DP0591	*Pyrus pyrifolia*	New Zealand	JQ807465	KJ420821	KJ435018	JQ807395
	DP0666	*Juglans cinerea*	USA	KJ210522	KJ420788	KJ435007	KJ210546
	FAU483	*Malus* sp.	Netherlands	KJ210537	KJ420827	KJ435022	KJ210556
	FAU522	*Sassafras albidum*	USA	KJ210525	KJ420791	KJ435010	JQ807406
	FAU532	*Chamaecyparis thyoides*	USA	JQ807333	KJ420815	KJ435015	JQ807408
	LCM11401b	*Ulmus* sp.	USA	KJ210520	KJ420786	KJ435026	KJ210544
	LCM11401	*Ulmus* sp.	USA	KJ210521	KJ420787	KJ435027	KJ210545
	M1118	*Vitis vinifera*	France	KJ210519	KJ420784	KJ434997	KJ210543
	M1115	*Daphne laureola*	France	KJ210516	KJ420781	KJ434994	KJ210540
	MAFF625033	*Pyrus pyrifolia*	Japan	JQ807468	KJ420814	KJ435017	JQ807417
	MAFF625034	*Pyrus pyrifolia*	Japan	JQ807469	KJ420819	KJ435023	JQ807418
*D. eucalyptorum*	CBS 132525	*Eucalyptus* sp.	Australia	NR120157	N/A	N/A	N/A
*D. foeniculacea*	CBS 123208	*Foeniculum vulgare*	Portugal	KC343104	KC344072	KC343346	KC343830
*D. fraxini- angustifoliae*	BRIP 54781	*Fraxinus angustifolia*	Australia	JX862528	KF170920	N/A	JX862534
*D. fraxinicola*	CFCC 52582	*Fraxinus chinensis*	China	MH121517	N/A	MH121435	MH121559
*D. fukushii*	MAFF 625034	*Pyrus pyrifolia*	Japan	JQ807469	N/A	N/A	JQ807418
*D. fusicola*	CGMCC 3.17087	*Lithocarpus glabra*	China	KF576281	KF576305	KF576233	KF576256
*D. ganjae*	CBS 180.91	*Cannabis sativa*	USA	KC343112	KC344080	KC343354	KC343838
*D. garethjonesii*	MFLUCC 12-0542a	*Unknown dead leaf*	Thailand	KT459423	KT459441	KT459470	KT459457
*D. goulteri*	BRIP 55657a	*Helianthus annuus*	Australia	KJ197290	KJ197270	N/A	KJ197252
*D. gulyae*	**BRIP 54025**	***Helianthus annuus***	**Australia**	**JF431299**	**JN645803**	**N/A**	**KJ197271**
*D. helianthi*	**CBS 592.81**	***Helianthus annuus***	**Serbia**	**KC343115**	**KC344083**	**KC343357**	**KC343841**
*D. helicis*	AR5211	*Hedera helix*	France	KJ210538	KJ420828	KJ435043	KJ210559
*D. heterophyllae*	CBS 143769	*Acacia heterohpylla*	France	MG600222	MG600226	MG600218	MG600224
*D. hickoriae*	CBS 145.26	*Carya glabra*	USA	KC343118	KC344086	KC343360	KC343844
*D. hispaniae*	**CPC 30321**	***Vitis vinifera***	**Spain**	**MG281123**	**MG281296**	**MG281820**	**MG281644**
*D. hongkongensis*	**CBS 115448**	***Dichroa febr**í**fuga***	**China**	**KC343119**	**KC344087**	**KC343361**	**KC343845**
*D.hungariae*	**CBS143353**	***Vitis vinifera***	**Hungary**	**MG281126**	**MG281299**	**MG281823**	**MG281647**
*D. incompleta*	CGMCC 3.18288	*Camellia sinensis*	China	KX986794	KX999226	KX999289	KX999186
*D. inconspicua*	CBS 133813	*Maytenus ilicifolia*	Brazil	KC343123	KC344091	KC343365	KC343849
*D. infecunda*	CBS 133812	*Schinus* sp.	Brazil	KC343126	KC344094	KC343368	KC343852
*D. isoberliniae*	CPC 22549	*Isoberlinia angolensis*	Zambia	KJ869133	KJ869245	N/A	N/A
	CFCC 51135	*Juglans mandshurica*	China	KU985102	KX024635	KX024617	KX024629
*D. kadsurae*	CFCC 52587	*Kadsura longipedunculata*	China	MH121522	MH121601	MH121440	MH121564
*D. kochmanii*	**BRIP 54033**	***Helianthus annuus***	**Australia**	**JF431295**	**N/A**	**N/A**	**JN645809**
*D. kochmanii*	BRIP 54034	*Helianthus annuus*	Australia	JF431296	N/A	N/A	JN645810
*D. kongii*	BRIP 54031	*Portulaca grandifl a*	Australia	JF431301	KJ197272	N/A	JN645797
*D. litchicola*	BRIP 54900	*Litchi chinensis*	Australia	JX862533	KF170925	N/A	JX862539
*D. lithocarpus*	CGMCC 3.15175	*Lithocarpus glabra*	China	KC153104	KF576311	KF576235	KC153095
*D. longicicola*	**CGMCC 3.17089**	***Lithocarpus glabra***	**China**	**KF576267**	**KF576291**	**N/A**	**KF576242**
	CGMCC 3 17090	*Lithocarpus glabra*	China	KF576268	KF576292	N/A	KF576243
*D. longispora*	CBS 194.36	*Ribes* sp.	Canada	KC343135	KC344103	KC343377	KC343861
*D. lonicerae*	MFLUCC 17-0963	*Lonicera* sp.	Italy	KY964190	KY964073	KY964116	KY964146
*D. lusitanicae*	CBS 123212	*Foeniculum vulgare*	Portugal	KC343136	KC344104	KC343378	KC343862
*D. macinthoshii*	BRIP 55064a	*Rapistrum rugostrum*	Australia	KJ197289	KJ197269	N/A	KJ197251
*D. mahothocarpus*	**CGMCC 3.15181**	***Lithocarpus glabra***	**China**	**KC153096**	**KF576312**	**N/A**	**KC153087**
*D. malorum*	CAA734	*Malus domestica*	Portugal	KY435638	KY435668	KY435658	KY435627
*D.momicola*	**MFLUCC 16-0113**	***Prunus persica***	**Hubei, China**	**KU557563**	**KU557587**	**KU557611**	**KU557631**
*D. maritima*	DAOMC 250563	*Picea rubens*	Canada	N/A	KU574616	N/A	N/A
*D. masirevicii*	BRIP 57892a	*Helianthus annuus*	Australia	KJ197277	KJ197257	N/A	KJ197239
*D. mayteni*	CBS 133185	*Maytenus ilicifolia*	Brazil	KC343139	KC344107	KC343381	KC343865
*D. maytenicola*	CPC 21896	*Maytenus acuminata*	South Africa	KF777157	KF777250	N/A	N/A
*D. melonis*	CBS 507.78	*Cucumis melo*	USA	KC343142	KC344110	KC343384	KC343868
*D. middletonii*	BRIP 54884e	*Rapistrum rugostrum*	Australia	KJ197286	KJ197266	N/A	KJ197248
*D. miriciae*	BRIP 54736j	*Helianthus annuus*	Australia	KJ197282	KJ197262	N/A	KJ197244
*D. multigutullata*	ZJUD98	*Citrus grandis*	China	KJ490633	KJ490454	N/A	KJ490512
*D. musigena*	CBS 129519	*Musa* sp.	Australia	KC343143	KC344111	KC343385	KC343869
*D. neilliae*	CBS 144.27	*Spiraea* sp.	USA	KC343144	KC344112	KC343386	KC343870
*D. neoarctii*	CBS 109490	*Ambrosia trifi*	USA	KC343145	KC344113	KC343387	KC343871
*D.neoraonikayaporum*	MFLUCC 14-1136	*Tectona grandis*	Thailand	KU712449	KU743988	KU749356	KU749369
*D. nobilis*	CBS 113470	*Castanea sativa*	Korea	KC343146	KC344114	KC343388	KC343872
*D. nothofagi*	BRIP 54801	*Nothofagus cunninghamii*	Australia	JX862530	KF170922	N/A	JX862536
*D. novem*	**CBS 127270**	***Glycine max***	**Croatia**	**KC343155**	**KC344123**	**KC343397**	**KC343881**
*D. ocoteae*	CBS 141330	*Ocotea obtusata*	France	KX228293	KX228388	N/A	N/A
*D. oraccinii*	CGMCC 3.17531	*Camellia sinensis*	China	KP267863	KP293443	N/A	KP267937
*D. ovalispora*	ICMP20659	*Citrus limon*	China	KJ490628	KJ490449	N/A	KJ490507
*D. ovoicicola*	CGMCC 3.17093	*Citrus* sp.	China	KF576265	KF576289	KF576223	KF576240
*D. oxe*	CBS 133186	*Maytenus ilicifolia*	Brazil	KC343164	KC344132	KC343406	KC343890
*D. padina*	CFCC 52590	*Padus racemosa*	China	MH121525	MH121604	MH121443	MH121567
	CFCC 52591	*Padus racemosa*	China	MH121526	MH121605	MH121444	MH121568
*D. pandanicola*	MFLU 18-0006	*Pandanus* sp.	Thailand	MG646974	MG646930	N/A	N/A
*D. paranensis*	CBS 133184	*Maytenus ilicifolia*	Brazil	KC343171	KC344139	KC343413	KC343897
*D. parapterocarpi*	CPC 22729	*Pterocarpus brenanii*	Zambia	KJ869138	KJ869248	N/A	N/A
*D. pascoei*	BRIP 54847	*Persea americana*	Australia	JX862532	KF170924	N/A	JX862538
*D. passifl ae*	CBS 132527	*Passifl a edulis*	South America	JX069860	N/A	N/A	N/A
*D. passifl*	CBS 141329	*Passifl a foetida*	Malaysia	KX228292	KX228387	N/A	N/A
*D. penetriteum*	CGMCC 3.17532	*Camellia sinensis*	China	KP714505	KP714529	N/A	KP714517
*D. perjuncta*	**CBS 109745**	***Ulmus glabra***	**Austria**	**KC343172**	**KC344140**	**KC343414**	**KC343898**
*D. perseae*	CBS 151.73	*Persea gratissima*	Netherlands	KC343173	KC344141	KC343415	KC343899
*D. pescicola*	**MFLU 16-0105**	***Prunus persica***	**Hubei, China**	**KU557555**	**KU557579**	**KU557603**	**KU557623**
*D. phaseolorum*	AR4203	*Phaseolus vulgaris*	USA	KJ590738	KP004507	N/A	N/A
*D.phragmitis*	**CBS 138897**	***Phragmites australis***	**China**	**KP004445**	**KP004507**	**N/A**	**N/A**
*D. podocarpi- macrophylli*	CGMCC 3.18281	*Podocarpus macrophyllus*	China	KX986774	KX999207	KX999278	KX999167
*D. pseudomangiferae*	CBS 101339	*Mangifera indica*	Dominican Republic	KC343181	KC344149	KC343423	KC343907
*D.pseudophoenicicola*	**CBS 462.69**	***Phoenix dactylifera***	**Spain**	**KC343184**	**KC344152**	**KC343426**	**KC343910**
*D. pseudotsugae*	MFLU 15-3228	*Pseudotsuga menziesii*	Italy	KY964225	KY964108	KY964138	KY964181
*D. psoraleae*	CBS 136412	*Psoralea pinnata*	South Africa	KF777158	KF777251	N/A	KF777245
*D. psoraleae- pinnatae*	CBS 136413	*Psoralea pinnata*	South Africa	KF777159	KF777252	N/A	N/A
*D. pterocarpi*	MFLUCC 10-0571	*Pterocarpus indicus*	Thailand	JQ619899	JX275460	JX197451	JX275416
*D. pterocarpicola*	MFLUCC 10-0580	*Pterocarpus indicus*	Thailand	JQ619887	JX275441	JX197433	JX275403
*D. pulla*	**CBS 338.89**	***Hedera helix***	**Yugoslavia**	**KC343152**	**KC344120**	**KC343394**	**KC343878**
*D. pyracanthae*	CAA483	*Pyracantha coccinea*	Portugal	KY435635	KY435666	KY435656	KY435625
*D. racemosae*	CBS 143770	*Euclea racemosa*	South Africa	MG600223	MG600227	MG600219	MG600225
*D. raonikayaporum*	CBS 133182	*Spondias mombin*	Brazil	KC343188	KC344156	KC343430	KC343914
*D. ravennica*	MFLUCC 15-0479	*Tamarix* sp.	Italy	KU900335	KX432254	N/A	KX365197
*D. rhusicola*	CBS 129528	*Rhus pendulina*	South Africa	JF951146	KC843205	KC843124	KC843100
*D. rosae*	MFLU 17-1550	*Rosa* sp.	Thailand	MG828894	MG843878	N/A	N/A
*D. rosicola*	MFLU 17-0646	*Rosa* sp.	UK	MG828895	MG843877	N/A	MG829270
*D. rostrata*	CFCC 50062	*Juglans mandshurica*	China	KP208847	KP208855	KP208849	KP208853
*D. rudis*	AR3422	*Laburnum anagyroides*	Austria	KC843331	KC843177	KC843146	KC843090
*D. saccarata*	**CBS 116311**	***Protea repens***	**South Africa**	**KC343190**	**KC344158**	**KC343432**	**KC343916**
*D. sackstonii*	BRIP 54669b	*Helianthus annuus*	Australia	KJ197287	KJ197267	N/A	KJ197249
*D. salicicola*	BRIP 54825	*Salix purpurea*	Australia	JX862531	JX862531	N/A	JX862537
*D. sambucusii*	CFCC 51986	*Sambucus williamsii*	China	KY852495	KY852511	KY852499	KY852507
*D. schini*	**CBS 133181**	***Schinus terebinthifolius***	**Brazil**	**KC343191**	**KC344159**	**KC343433**	**KC343917**
*D. schisandrae*	CFCC 51988	*Schisandra chinensis*	China	KY852497	KY852513	KY852501	KY852509
*D. schoeni*	MFLU 15-1279	*Schoenus nigricans*	Italy	KY964226	KY964109	KY964139	KY964182
*D. sclerotioides*	CBS 296.67	*Cucumis sativus*	Netherlands	KC343193	KC344161	KC343435	KC343919
*D. sennae*	CFCC 51636	*Senna bicapsularis*	China	KY203724	KY228891	KY228875	KY228885
*D. sennicola*	CFCC 51634	*Senna bicapsularis*	China	KY203722	KY228889	KY228873	KY228883
*D. serafi*	BRIP 55665a	*Helianthus annuus*	Australia	KJ197274	KJ197254	N/A	KJ197236
*D. siamensis*	MFLUCC 10-573a	*Dasymaschalon* sp.	Thailand	JQ619879	JX275429	N/A	JX275393
*D. sojae*	**FAU635**	***Glycine max***	**Ohio, USA**	**KJ590719**	**KJ610875**	**KJ612116**	**KJ590762**
	BRIP 54033	*Helianthus annuus*	Australia	JF431295	KJ160528	KJ160548	JN645809
	CBS116019	*Caperonia palustris*	USA	KC343175	KJ610862	KJ612103	KC343901
	DP0601	*Glycine max*	USA	KJ590706	N/A	N/A	KJ590749
	DP0605	*Glycine max*	USA	KJ590707	KJ610863	KJ612104	KJ590750
	DP0616	*Glycine max*	USA	KJ590715	KJ610871	KJ612112	KJ590758
	FAU455	*Stokesia laevis*	USA	KJ590712	KJ610870	KJ612111	KJ590755
	FAU458	*Stokesia laevis*	USA	KJ590710	KJ610866	KJ612107	KJ590753
	FAU459	*Stokesia laevis*	USA	KJ590709	KJ610865	KJ612106	KJ590752
	FAU499	*Asparagus officinalis*	USA	KJ590717	KJ610873	KJ612114	KJ590760
	FAU604	*Glycine max*	USA	KJ590716	KJ610872	KJ612113	KJ590759
	FAU636	*Glycine max*	USA	KJ590718	KJ610874	KJ612115	KJ590761
	ZJUD68	*Glycine max*	USA	KJ490603	KJ490424	**N/A**	KJ490482
	ZJUD69	*Citrus reticulata*	China	KJ490604	KJ490425	**N/A**	KJ490483
	ZJUD70	*Citrus limon*	China	KJ490605	KJ490426	**N/A**	KJ490484
*D. spartinicola*	CBS 140003	*Spartium junceum*	Spain	KR611879	KC344180	KC343454	N/A
*D. sterilis*	CBS 136969	*Vaccinium corymbosum*	Italy	KJ160579	KJ490408	N/A	KJ160611
*D. stictica*	CBS 370.54	*Buxus sampervirens*	Italy	KC343212	MG746631	N/A	KC343938
*D. subclavata*	**ICMP20663**	***Citrus unshiu***	**China**	**KJ490587**	**MG746634**	**N/A**	**KJ490466**
*D. subcylindrospora*	MFLU 17-1195	*Salix* sp.	China	MG746629	KC344182	KC343456	MG746630
*D. subellipicola*	**MFLU 17-1197**	**on dead wood**	**China**	**MG746632**	**KU557591**	**KU557567**	**MG746633**
*D. subordinaria*	CBS 464.90	*Plantago lanceolata*	New Zealand	KC343214	KU557592	KU557568	KC343940
*D. taoicola*	**MFLUCC 16 0117**	***Prunus persica***	**Hubei, China**	**NR154923**	**KU743977**	**KU712430**	**KU557635**
*D. tectonae*	MFLUCC 12 0777	*Tectona grandis*	Thailand	NR147590	KU743977	KU749345	KU749359
*D. tectonigena*	MFLUCC 12-0767	*Tectona grandis*	China	KU712429	JX275449	JX197440	KU749371
*D. terebinthifolii*	**CBS 133180**	***Schinus terebinthifolius***	**Brazil**	**KC343216**	**N/A**	**N/A**	**KC343942**
*D. thunbergii*	MFLUCC 10-576a	*Th laurifolia*	Thailand	JQ619893	MF279873	MF279888	JX275409
*D. thunbergiicola*	MFLUCC 12-0033	*Th laurifolia*	Thailand	KP715097	MF279874	MF279889	KP715098
*D. tibetensis*	CFCC 51999	*Juglandis regia*	China	MF279843	KY964096	KY964127	MF279858
*D. torilicola*	MFLUCC 17-1051	*Torilis arvensis*	Italy	KY964212	KR936132	N/A	KY964168
*D. toxica*	**CBS 534.93**	***Lupinus angustifolius***	**Australia**	**KC343220**	**KJ610881**	**KJ612122**	**KC343946**
*D. tulliensis*	**BRIP62248a**	***Theobroma cacao***	**Australia**	**KR936130**	**N/A**	**MH121445**	**KR936133**
*D. ueckerae*	FAU656	*Cucumis melo*	USA	KJ590726	N/A	MH121446	KJ590747
*D. ukurunduensis*	CFCC 52592	*Acer ukurunduense*	China	MH121527	KX999230	N/A	MH121569
	**CFCC 52593**	***Acer ukurunduense***	**China**	**MH121528**	**KJ490408**	**N/A**	**MH121570**
*D. undulata*	CGMCC 3.18293	*Leaf of unknown host*	China-Laos border	KX986798	KJ490406	N/A	KX999190
*D. unshiuensis*	ZJUD50	*Fortunella margarita*	China	KJ490585	KC344195	KC343469	KJ490464
*D. vaccini*	**CBS160 32**	***Oxycoccus macrocarpos***	**USA**	**KC343228**	KJ869247	N/A	**KC343954**
*D. vangueriae*	CPC 22703	*Vangueria infausta*	Zambia	KJ869137	KX999223	N/A	N/A
*D. vawdreyi*	BRIP 57887a	*Psidium guajava*	Australia	KR936126	KP247575	N/A	KR936129
*D. velutina*	CGMCC 3.18286	*Neolitsea* sp.	China	KX986790	KX999216	N/A	KX999182
*D. virgiliae*	CMW40748	*Virgilia oroboides*	South Africa	KP247566	KX999228	KX999290	N/A
*D. xishuangbanica*	CGMCC 3.18282	*Camellia sinensis*	China	KX986783	KC343972	KC343246	KX999175
*D. yunnanensis*	CGMCC 3.18289	*Coff* sp.	China	KX986796	N/A	KX999290	KX999188
*Diaporthella corylina*	**CBS 121124**	***Corylus*** **sp**.	**China**	**KC343004**	**KC343972**	**KC343246**	**KC343730**

*BRIP, Plant Pathology Herbarium, Department of Primary Industries, Dutton Park, Queensland, Australia; CPC, Culture collection of P.W. Crous, housed at Westerdijk Fungal Biodiversity Institute; CBS, Westerdijk Fungal Biodiversity Institute, Utrecht, The Netherlands; DAOM, Canadian Collection of Fungal Cultures or the National Mycological Herbarium, Plant Research Institute, Department of Agriculture (Mycology), Ottawa, Canada; ICMP, International Collection of Microorganisms from Plants, Landcare Research, Auckland, New Zealand. MFLUCC, Mae Fah Luang University culture collection, Mae Fah Luang University, Chiang Rai, 57100, Thailand. JZB, Culture collection of Institute of Plant and Environment Protection, Beijing Academy of Agriculture and Forestry Sciences, Beijing 100097, China. AR, DAN, DNP, FAU, DLR, DF, DP, LCM, M, isolates in SMML culture collection, USDA-ARS, Beltsville, MD, USA, and MAFF, NIAS Genebank Project, Ministry of Agriculture, Forestry and Fisheries, Japan. Ex-type and ex-epitype cultures are indicated in bold. ITS, internal transcribed spacers 1 and 2 together with 5.8S nrDNA; β-tubulin, partial beta-tubulin gene; CAL, partial calmodulin gene and EF-1α, partial translation elongation factor 1-α gene*.

In PAUP, ambiguous regions in the alignment were excluded for further analyses, and gaps were treated as missing data. The stability of the trees was evaluated by 1000 bootstrap replications. Branches of zero length were collapsed, and all multiple parsimonious trees were saved. Parameters, including tree length (TL), consistency index (CI), retention index (RI), relative consistency index (RC), and homoplasy index (HI) were calculated. Differences between the trees inferred under different optimality criteria were evaluated using Kishino-Hasegawa tests (KHT) (Kishino and Hasegawa, 1989). The evolutionary models for each locus used in Bayesian analysis and ML were selected using MrModeltest v. 2.3 (Nylander, 2004). ML analyses were accomplished using RAxML-HPC2 on XSEDE (8.2.8) (Stamatakis et al., 2008; Stamatakis, 2014) in the CIPRES Science Gateway platform (Miller et al., 2010) using the GTR + I + G model of evolution with 1000 non-parametric bootstrapping iterations. Bayesian analysis was performed in MrBayes v. 3.0b4 (Ronquist and Huelsenbeck, 2003), and posterior probabilities (PPs) were determined by Markov chain Monte Carlo sampling (MCMC). Six simultaneous Markov chains were run for 106 generations, sampling the trees at every 100th generation. From the 10,000 trees obtained, the first 2,000 representing the burn-in phase were discarded. The remaining 8,000 trees were used to calculate PPs in a majority rule consensus tree. Alignment generated in this study is submitted to TreeBASE (https://treebase.org/treebase-web/home.html) under the submission number 24324. Taxonomic novelties were submitted to the Faces of Fungi database (Jayasiri et al., 2015) and Index fungorum (http://www.indexfungorum.org). New species are described following Jeewon and Hyde (2016).

### Morphology and Culture Characteristics

Colony morphology and conidial characteristics were examined for *Diaporthe* species identified by phylogenetic analysis. Colony colors were examined according to Rayner (1970) after 7 days of growth on PDA in the dark at 25°C. Digital images of morphological structures mounted in water were taken using an Axio Imager Z2 photographic microscope (Carl Zeiss Microscopy, Oberkochen, Germany). Measurements were taken using ZEN PRO 2012 (Carl Zeiss Microscopy). Conidial length and width were measured for 40 conidia per isolate, and the mean values were calculated for all measurements. Conidial shape, color, and guttulation were recorded.

### Genetic Diversity and Population Structure Analysis

Among the identified species, only one, *Diaporthe eres*, had a count of >20 individuals. As a result, only *D. eres* was selected for the analysis of genetic diversity and population relationships. For the *D. eres* population, diversity indices were calculated for each gene region and the combined sequence dataset. DnaSP v. 6.12 (Librado and Rozas, 2009) was employed to calculate haplotype richness (hR), the total number of haplotypes, Watterson's theta (Θw), and pairwise nucleotide diversity (JI). To overcome the population size effects, hR, Θw and JI were calculated after 1,000 repetitions, and the median estimate was recorded for each parameter. To understand the potential departure from an equilibrium model of evolution, Tajima's D was calculated using DnaSP v. 6.12 with a permutation test of 1,000 replicates. The minimum numbers of recombination events (ZnS) used by Kelly (1997) and the recombination parameters Za and ZZ used by Hudson (1983) were calculated for each gene region and the combined data set. *Diaporthe eres* haplotype networks were constructed using Network v. 5.0 (Bandelt et al., 1999).

### Network Analysis

To understand the relationship among different geographical populations, recombination parameters were calculated, and haplotype networks were constructed. In this analysis, the combined dataset of *Diaporthe eres* isolates from China alone and Chinese isolates combined with European isolates (Guarnaccia et al., 2018) were used. ZnS, used by Kelly (1997), and the recombination parameters Za and ZZ (Hudson, 1983; Kelly, 1997) were calculated using DnaSP v. 6.12. The haplotype data generated using DnaSP v. 6 were used to construct a median-joining network in Network v. 5.0 (Bandelt et al., 1999).

### Pathogenicity Assay

The pathogenicity and aggressiveness of the *Diaporthe* species were tested using detached green shoots of the *V. vinifera* cultivar Summer Black. Healthy, 30–50 cm long green shoots (including at least two nodes) were obtained from “Shunyi Xiangyi” vineyard in Beijing, China, where *Diaporthe* species were not recorded. The cuttings were surface-sterilized with 70% ethanol by wiping with cotton swabs. A shallow wound (5 mm length, 2 mm deep) was made in the center of each shoot using a sterilized scalpel. Mycelial plugs were taken from the growing margin of a 5-day-old culture grown in PDA and inoculated at the wound site. Non-colonized sterile PDA plugs were used for inoculation of shoots as a negative control. To prevent drying, all inoculated areas were covered with Para-film (Bemis, USA). Inoculated shoots were kept in a growth chamber for 21 days at 25°C with a 12 h photoperiod. The experiment was organized with 10 replicates for each isolate. Pathogenicity test was repeated three times with same controlled environment. A total of 16 strains from eight species were tested. The presence of lesions advancing beyond the original 0.5 cm diameter inoculation point was considered indicative of pathogenicity. The experimental design was completely randomized. Data were analyzed with a one-way ANOVA (analysis of variance) using Minitab v. 16.0 (Minitab Inc., Boston, MA, USA), with statistical significance set at the 5% level. The pathogens were re-isolated to confirm their identity.

## Results

### Initial Species Identification and Phylogenetic Analyses

During our field survey on six grape-growing provinces in China (Figure 1), we collected samples with typical symptoms associated with Diaporthe dieback, such as wedge-shaped cankers, and light brown streaking of the wood (Figure 2). However, these symptoms are sometimes confused with other grape trunk disease symptoms caused by Botryosphaeria dieback, Eupta, and Esca (Mondello et al., 2018). Hence, further confirmation is required by isolating and identifying causal organisms. One hundred and eleven *Diaporthe* isolates were initially identified by colony characteristics, such as abundant tufted white aerial mycelia on agar medium. The ITS gene regions were sequenced for all fungi isolated from diseased shoots and compared with those in GenBank using the MegaBLAST tool in GenBank. The isolates showed 95–99% similarity to known *Diaporthe* species in GenBank, and these closely related known species were included in the phylogenetic analysis.

To understand the taxonomic placements of our isolates, additional gene regions, including those encoding EF-1α, β-tubulin, and CAL, were sequenced. Then, phylogenetic trees were constructed for each individual gene region. The concatenated sequence data set consisted of 94 isolates (out of 111, due to sequencing errors) from the current study (Table 3) and 197 isolates originating from GenBank (Table 2), with one outgroup taxon, *Diaporthella corylina* (CBS 121124). A comparison of maximum likelihood (ML) analysis results for each gene region is given in Table 4. In the ML analysis, the resulting tree of the combined data set of ITS, β-tubulin, CAL, and EF-1α genes had the best resolution of taxa (Figure 3). Therefore, in the present study, we used the combined sequence data to understand the taxonomic placements of the *Diaporthe* species isolated from grapevines in China. A Bayesian analysis resulted in 10,001 trees after 2,000,000 generations. The first 1,000 trees, representing the burn-in phase of the analyses, were discarded, while the remaining 9,001 trees were used for calculating posterior probabilities (PPs) in the majority-rule consensus tree. The dataset consisted of 1,494 characters with 727 constant characters and 1,006 parsimony-informative and 213 parsimony-uninformative characters. The maximum number of trees generated was 1,000, and the most parsimonious trees had a tree length of 9,862 (CI = 0.249, RI = 0.805, RC = 0.201, HI = 0.751).

**Table 4:** Comparison of ML analyses results for each gene region.

**Data set**	**ITS**	**β-tubulin**	**CAL**	**EF-1α**	**ITS+ β-tubulin+ CAL+ EF-1α**
Constant characters	226	226	226	68	
Parsimony-uninformative characters	107	26	107	48	
Parsimony-informative characters	189	249	189	335	
ML optimization likelihood value	−51,581.507970	−9741.212701	−7853.669691	−16943.655728	−50,588.257001
Distinct alignment patterns	291	304	293	293	1,330
Undetermined characters or gaps	7.18%	26.12%	8.74%	28.55%	28.70%
**ESTIMATED BASE FREQUENCIES**					
A	0.244043	0.200039	0.211490	0.220112	0.221742
C	0.277339	0.349071	0.313694	0.329420	0.313804
G	0.247357	0.233934	0.253908,	0.250506	0.235189
T	0.231261	0.216955	0.220908	0.220908	0.229264
**SUBSTITUTION RATES**					
AC	1.300271	0.791706	1.041213	1.457977	1.328496
AG	2.994990	3.761550	4.289330	3.778337	3.630252
AT	1.401626	0.962021	1.307157	1.339450	1.324920
CG	0.826919	0.668475	1.259772	1.119872	0.954109
CT	7.266633	7.266633	5.662938	3.976963	4.974568
GT	1.000000	1.000000	1.000000	1.000000	1.000000
Proportion of invariable sites (I)	0.274443	0.350656	0.274443	0.274443	0.269146
Gamma distribution shape parameter (α)	0.405766	2.208572	0.405766	0.405766	0.869283

In the phylogenetic tree generated using the combined data set (Figure 3), 36 isolates from the present study clustered with *Diaporthe eres* in the *D. eres* complex. This group represents 37.5% of the total isolates, and these isolates were obtained from five provinces. Sixteen isolates (19.76% of the total isolates) clustered with *Diaporthe sojae* (*D. sojae*) species in the *D. sojae* complex. Two isolates from Heilongjiang province clustered together with *Diaporthe gulyae* (*D. gulyae)* (BRIP 54025). In addition, two isolates clustered with *Diaporthe unshiuensis* (*D. unshiuensis)* (ZJUD52) from Hubei province, and another two isolates that were also from Hubei province clustered with *Diaporthe pescicola* (*D. pescicola)* (MFLUCC 16-0105). The remaining isolates (35 in total) did not cluster with any known *Diaporthe* species. Thus, these were putatively identified as belonging to three novel species (Figure 3): *D. hubeiensis, D. guangxiensis*, and *D. viniferae*. *Diaporthe hubeiensis (D. hubeiensis)* was isolated from grapevines from Hubei province and represents 12.5% of the total isolates. This species is a sister taxon with *Diaporthe alangi (D. alangi)* (CFCC52556). The remaining two new taxa were isolated from grapevines from Guangxi Province. *Diaporthe guangxiensis* (*D. guangxiensis)* was represented by 11 isolates (13.54%), and it is closely associated with *Diaporthe cercidis (D. cercidis)* (CFCC5255). *Diaporthe viniferae (D. viniferae)* was represented by 8 isolates (10.41%), and its closest relative is *Diaporthe pandanicola (D. pandanicola)* (MFLU 18-0006).

### Taxonomic Novelties

**Figure 4:**
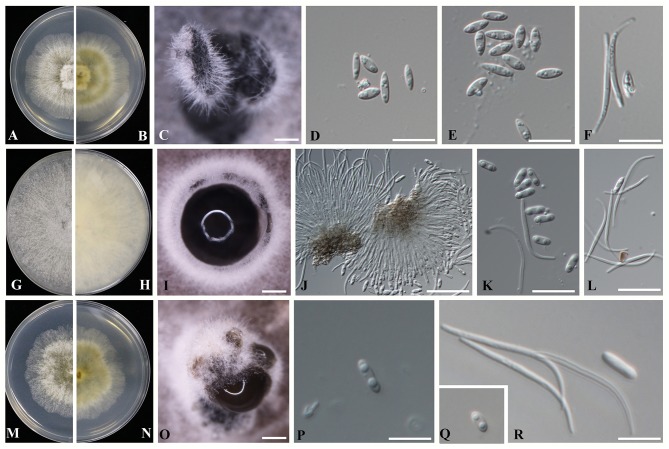
Novel *Diaporthe* taxa identified in the present study **(A–F)** *Diaporthe guangxiensis* **(A,B)** Culture on PDA after 5 days; **(C)** Pycnidia on PDA; **(D,E)** Alpha conidia; and **(F)** Beta conidia. **(G–L)** *Diaporthe hubeiensis* **(G,H)** Culture on PDA after 5 days; **(I)** Pycnidia on PDA; **(J)** Conidiogenous cells for alpha and beta conidia; **(K)** Alpha conidia, and **(L)** Beta conidia. **(M–R)** *Diaporthe viniferae* **(M,N)** Culture on PDA after 5 days; **(O)** Pycnidia on PDA; **(P,Q)** Alpha conidia; and **(R)** Beta conidia. Scale bars: **(D–F,J–L,P–R)** = 1 mm; **(C,I,O)** = 10 μm.

***Diaporthe guangxiensis*** *(D. guangxiensis)* Dissanayake, X.H. Li & K.D. Hyde, **sp**. ***nov***. (Figure 4).

*Index Fungorum number*—IF552578, *Facesoffungi Number*- FoF02725.

Etymology- In reference to the Guangxi Province, from where the fungus was first isolated.

Holotype—JZBH320094.

#### Description

Sexual morph: efforts were made to initiate sexual morphs, but various methods failed; Asexual morph: pycnidia on PDA 250-1550 μm (*x* = 1100 μm, *n* = 20) in diam., superficial, scattered on PDA, dark brown to black, globose, solitary, or clustered in groups of 3–5 pycnidia. Conidiophores aseptate, cylindrical, straight or sinuous, densely aggregated, terminal, slightly tapered toward the apex, 21–35 × 1.5–2.5 μm ($\bar{x}$ = 27 × 2 μm). Alpha conidia biguttulate, hyaline, fusiform or oval, both ends obtuse 5.3–7.8 × 1.5–3.2 μm ($\bar{x}$ = 6.8 × 2.5 μm *n* = 40). Beta conidia aseptate, hyaline, hamate, filiform, guttulate, tapering toward both ends 20–32 × 1–1.5 μm ($\bar{x}$ = 27 × 1.5 μm, *n* = 20).

#### Culture Characteristics

Colonies on PDA reach 70 mm diam. after 7 days at 25°C, producing abundant white aerial mycelia and reverse fuscous black.

#### Material Examined

CHINA, Guangxi Province, Pingguo County, on diseased trunk of *V. vinifera*, 3 June 2015, X.H. Li, (JZBH320094, holotype); ex-type living cultures JZB320094).

*Notes*: Morphological characters such as spores and colony characteristics of *D. guangxiensis* fit well within the species concept of *Diaporthe*. DNA sequence analyses of the ITS, CAL, TUB, and EF genes showed a strongly supported monophyletic lineage with 78% ML, 70% MP bootstrap values and 0.95 posterior probabilities (Figure 3). The current species has a particular neighbor relationship with *D. cercidis* (CFCC52566). Morphologically, *D. guangxiensis* has larger conidiophores (27 × 2 μm) and smaller conidia (6.8 × 2.5 μm) than *D. cercidis* (7–17 × 1.4–2.1 μm conidiophores; 8.6 × 3.3 μm conidia) (Yang et al., 2018). In the comparisons of five gene regions between *Diaporthe guangxiensis* and *D. cercidis*, 51.5% of 458 nucleotides across the ITS (+5.8S) had base pair differences. In addition, comparisons of the protein-coding genes showed that there were 17.3, 0.66, and 9.06% polymorphic nucleotide sites between the two species for the CAL, β-tubulin and EF-1α genes, respectively.

***Diaporthe hubeiensis*** Dissanayake, X.H. Li & K.D. Hyde, **sp**. ***nov***. (Figure 4).

*Index Fungorum number*—IF552579, *Facesoffungi Number*- FoF 02726.

Etymology- In reference to the Hubei province, from where the fungus was first isolated.

Holotype – JZBH320123.

#### Description

Sexual morph: efforts were made to initiate sexual morphs, but various methods failed; Asexual morph: pycnidia on PDA varying in size up to 510 μm in diam., subglobose, occurs on PDA and double-autoclaved toothpicks after 3–4 weeks, solitary or forms in groups of stroma with a blackened margin. Ostiolate, up to 100 μm black cylindrical necks. Conidiophores were reduced to conidiogenous cells. Alpha conidia hyaline, smooth, biguttulate, blunt at both ends, ellipsoidal to cylindrical, 5.6–7.1 × 1–3.1 μm ($\bar{x}$ = 6.1 × 1.8 μm *n* = 40). Beta conidia filiform, tapering toward both ends, scattered among the alpha conidia 17–27 × 1–1.5 μm ($\bar{x}$ = 24 × 1.5 μm *n* = 40).

#### Culture Characteristics

Colonies on PDA reach 90 mm after 10 days at 25°C (covers total surface), abundant tufted white aerial mycelia, buff, numerous black pycnidia 0.5 mm in diam. occur in the mycelium, typically in the direction of the edge of the colony; reverse buff with concentric lines.

#### Material Examined

CHINA Hubei Province, Wuhan, on diseased trunk of *V. vinifera*, 30 June 2015, X. H Li (JZBH320123, holotype); ex-type living cultures JZB320123.

*Notes*: In phylogenetic analysis, *D. hubeiensis* was placed in a well-supported clade together with *D. alangi* (CFCC52556), *D. tectonae* (MFLUCC 12- 0777) and *D. tulliensis* (BRIP62248b) with 100% ML, 100% MP bootstrap values and 0.99 posterior probabilities. *Diaporthe hubeiensis* developed sister clade with *D. alangi* (CFCC52556) with 99% ML, 83% MP bootstrap values and 0.99 posterior probabilities. Morphologically, *Diaporthe hubeiensis* has smaller conidiophores and smaller conidia (6.1 × 1.8 μm) than *D. alangi* (7 × 2 μm), and it has no beta conidia in *D. alangi* (Yang et al., 2018). *Diaporthe hubeiensis* differs from *D. tectonae* by developing wider but shorter conidia (6.1 × 1.8 μm vs 5.5 × 2.6 μm) (Doilom et al., 2017). Compared to *D. tulliensis, D. hubeiensis* has smaller conidia (6.1 × 1.8 μm vs 5.5–6 μm) (Yang et al., 2018). In the ITS sequence comparison between *D. hubeiensis* and *D. alangi*, 44.6% of the 461 nucleotides across the ITS (+5.8S) were different. Of the three protein-coding genes, the two species showed 4.26% and 1.16% and 5.3% polymorphic nucleotide site differences for CAL, β-tubulin and EF-1α genes, respectively.

***Diaporthe viniferae*** Dissanayake, X.H. Li & K.D. Hyde, ***sp. nov*.**

*Index Fungorum number*—IF552002, *Facesoffungi Number*- FoF 05981.

Etymology- In reference to the host *V. vinifera*.

Holotype—JZBH320071.

#### Description

Sexual morph: efforts were made to initiate sexual morphs, but various methods failed; Asexual morph: *Pycnidia* on PDA 363–937 μm (*x* = 529 μm, *n* = 20) in diam., superficial, scattered, dark brown to black, globose, solitary in most. Conidiophores were not observed. Conidiogenous cells were not observed. Alpha conidia biguttulate, hyaline, fusiform or oval, both ends obtuse 5–8.3 × 1.3–2.5 μm ($\bar{x}$ = 6.4 × 2.1 μm). Beta conidia aseptate, hyaline, hamate, filiform, tapering toward both ends 23–35 × 1–1.5 μm ($\bar{x}$ = 28 × 1.3 μm *n* = 40).

#### Culture Characteristics

Colonies on PDA reach 70 mm diam. after 7 days at 25°C, producing abundant white aerial mycelia and reverse fuscous black.

#### Material Examined

CHINA, Guangxi Province, Pingguo County, on the diseased trunk of *V. vinifera*, 3 June 2015, X.H. Li, (JZBH320071 holotype); ex-type living cultures JZB320071).

*Notes*: In the phylogenetic analysis of *D. viniferae*, a strongly supported monophyletic lineage with strong 77% ML and 71% MP bootstrap values and 0.95 PP was developed (Figure 3). The current species has a particular close relationship with *D. pandanicola* (MFLUCC 18-0006). In the original description of *D. pandanicola*, morphological characteristics were not given (Tibpromma et al., 2018). Therefore, these two species were compared based on only DNA sequence data. ITS sequence comparison between *D. viniferae* and *D. pandanicola* revealed that 2.9% of the 478 nucleotide sites across the ITS (+5.8S) regions were different. Similarly, 1.7% of the β-tubulin gene fragment was different.

### Genetic Diversity and Population Structure Analysis

**Figure 5:**
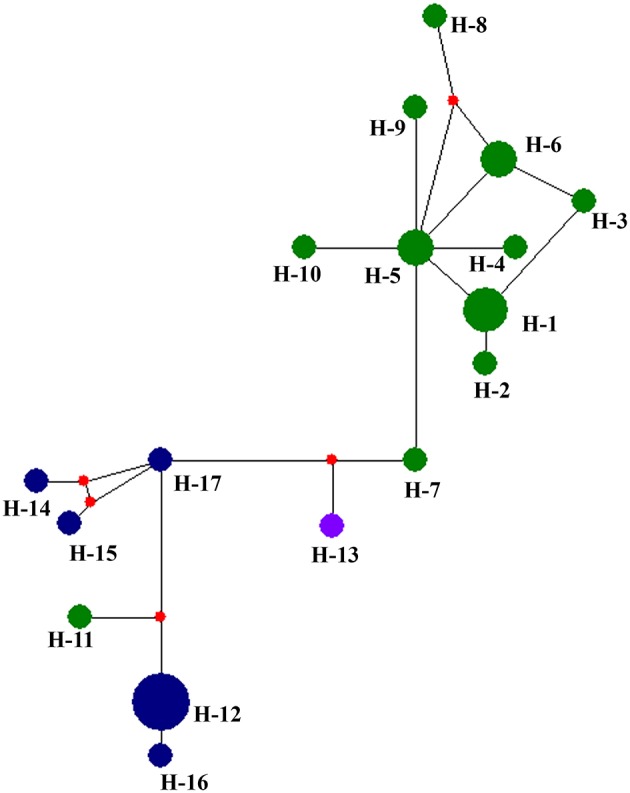
Haplotype network generated for the *Diaporthe eres* isolates obtained in the present study using Network v 6.0. At each node, sizes are propionate to the number of isolates. Blue, haplotypes from Jilin; Green, haplotypes from Hubei; purple, haplotypes from Sichuan; red, Median vectors.

Table 5 summarized the genetic diversity data of *D. eres* associated with grapevines which were estimated using DnaSP V.6. In the analysis, the combined data set of ITS, β-tubulin, HIS, APN, and CAL gene sequences showed 0.16226 segregation sites per sequence and a haplotype diversity of 0.955. A haplotype network was developed for the *D. eres* species isolated from China using Network v. 5.0 (Figure 5). The resulting network combining ITS, β-tubulin, HIS, EF-1α, and CAL gene sequences gave two main clusters according to geographic origin. In the network, isolates from Hubei province were clustered into two main clades. A single haplotype (H-11) was clustered within the main Jilin clade. Haplotype 7 (from Hubei) and h-13 (from Sichuan Province) were connected with one intermediate haplotype to the two main clusters.

**Table 5:** Polymorphism and genetic diversity of *Diaporthe eres* strains associated with Chinese grapevines.

**Species**	**Gene**	**n^a^**	**bp^b^**	**Theta-w**	**S^c^**	**h^d^**	**hd^e^**	**pi^f^**	**TD^g^**
*D. eres*	ITS	28	491	12.766	33	10	0.852	0.020	1.05556
	β-tubulin	28	481	6	26	10	0.869	0.01362	−0.35308
	HIS	15	244	0.04088	3	4	0.776	0.00167	−0.5791
	CAL	17	399	0.03590	15	11	0.845	0.01391	0.63457
	APN	16	680	0.00906	11	5	0.8	0.00445	−0.33503
	Combine	25	3247	0.01576	60	17	0.958	0.020	0.20416

a *Sample size (n)*.

b *Total number of sites (bp)*.

c *Number of segregating sites (S)*.

d *Number of alleles (nA)*.

e *Haplotypic (allelic) diversity (hd)*.

f *Average nucleotide diversity (pi)*.

g *Tajima's D (TD), (R) Estimate of R (Rm) minimum recombination events*.

**Figure 6:**
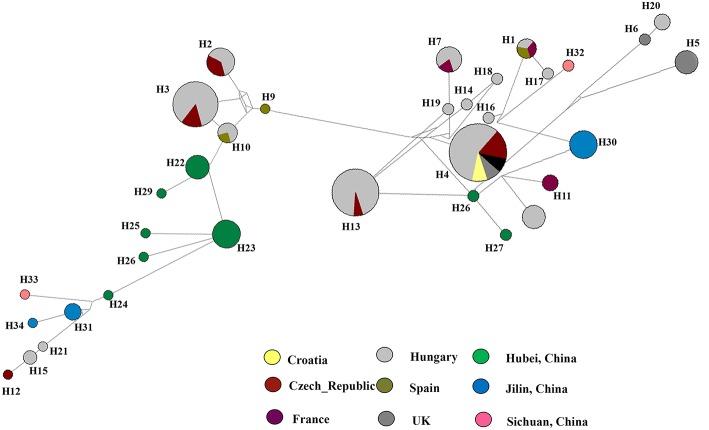
Haplotype network generated for the *Diaporthe eres* isolates from China and European countries using Network v 6.0. At each node, sizes are proportionate to the number of isolates.

To understand the relationship between *Diaporthe* isolates from Chinese vineyards and those from European vineyards, we calculated recombination parameters Z and ZnS. The combined data set consists of 135 sequences with 2203 sites. The estimate of R per gene was 6.6, and the minimum number of recombination events (Rm) was 15. Median-joining networks were constructed using both single-gene data files and a combined data set of ITS, β-tubulin, HIS, EF-1α, and CAL genes. The single-gene networks differed from each other, and the resulting patterns did not give a significant grouping. Therefore, in this study, only the combined network was considered (Figure 6). A total of 33 haplotypes were identified using DnaSP, and the haplotype data file was used to generate the haplotype network. In the resulting network, we found that Chinese haplotypes and Europe haplotypes were not shared and that there was no sharing of haplotypes among different provinces in China. However, the Chinese haplotypes were dispersed in the combined network, with the majority of isolates from Hubei located in two related clusters surrounded by European haplotypes. Similarly, the haplotypes from Sichuan and Jilin provinces were also dispersed in the network and close to both European and Chinese haplotypes.

### Comparative Aggressiveness Among *Diaporthe* Species

**Figure 7:**
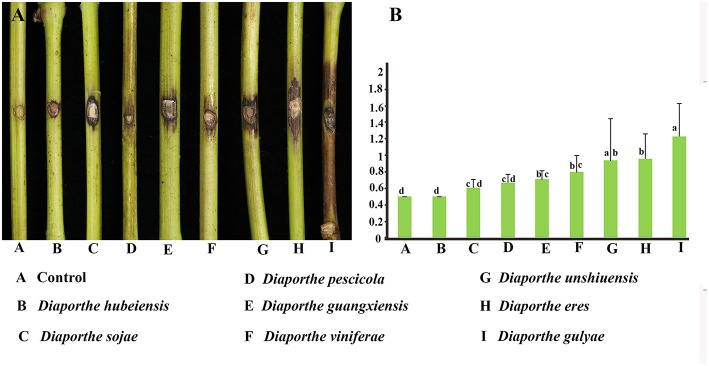
Pathogenicity test results for eight *Diaporthe* species associated with Chinese grapevines. **(A)** Variation in the development of lesions. **(B)** Mean lesion length (cm) at 21 days after inoculation of wounded detached healthy *Vitis vinifera* (*V. vinifera)* shoots (*n* = 10 per species).

Pathogenicity and aggressiveness among eight *Diaporthe* species isolated in our study were compared by inoculating them into the *V. vinifera* cultivar Summer Black. The inoculated shoots did not show significant lesion development within the first 2 weeks after inoculation. Brown necrotic lesions were detected both on the tissue surface and internally, advancing upwards, and downwards through the inoculation point. Twenty-one days after inoculation, *D. gulyae* developed the largest lesions (1.23 cm), followed by *D. eres* (0.94 cm). The remaining species*, D. unshiuensis, D. viniferae, D. guangxiensis, D. pescicola*, and *D. sojae*, exhibited similar levels of aggressiveness on grape shoots (Figure 7). *Diaporthe hubeiensis* was the least aggressive (0.5 cm) among the eight species.

## Discussion

Grapevine trunk disease has become one of the most devastating grapevine diseases in recent decades. According to data collected worldwide, ~1.5 billion US dollars per year is spent to replace dead grapevines due to these trunk diseases (Hofstetter et al., 2012; Fontaine et al., 2016). This is a great concern among grape-producing countries, as the disease infects perennial parts of the vine and reduces the productive lifespan of vines by several years (Gramaje and Armengol, 2011). The disease ultimately affects the sustainability of the wine industry and table grape production (Fontaine et al., 2016). As the world's top grape-producing country, China has strived to improve the quality and quantity of grapes. Though they are the most important grapevine trunk diseases worldwide, there is no evidence of either the esca complex or Eutypa dieback in China (Fontaine et al., 2016). However, the third most common grapevine trunk disease, caused by the species in *Botryosphaeriaceae* (Yan et al., 2013, 2018), has been identified as the leading grapevine trunk pathogen complex in China. Unfortunately, over the last few years, diseases caused by *Diaporthe* species (Dissanayake et al., 2015a, 2017) have become the emerging trunk diseases in China. Understanding the diversity of the causative species and the genetic variation within pathogen populations could help in developing sustainable disease management strategies. In addition, understanding the relationships between European and Chinese isolates can help track disease spread, as both regions share similar disease severity and *Diaporthe* species that differ from those in North America (Fontaine et al., 2016; Úrbez Torres and O'Gorman, 2019). To achieve these objectives, disease surveys were conducted in six provinces. We isolated and identified 111 *Diaporthe* strains and showed that they belong to eight species.

In 1958, *D. ampelina* (= *Phomopsis viticola*) was identified infecting green shoots of grapevines (Pscheidt and Pearson, 1989). The disease was named “Phomopsis cane and trunk disease.” According to the USDA Fungal—host interaction database, there are 166 records of *Diaporthe* species associated with grapevines worldwide (https://nt.ars-grin.gov/fungaldatabases/fungushost/fungushost.cfm) (Farr and Rossman, 2019). These records are related to the following 27 *Diaporthe* species: *Diaporthe ambigua (D. ambigua)* (Dissanayake et al., 2017), *D. ampelina* (Úrbez-Torres et al., 2013), *Diaporthe amygdali (D. amygdali)* (Gomes et al., 2013; Guarnaccia et al., 2018), *Diaporthe australafricana (D. australafricana)* (Gomes et al., 2013), *Diaporthe baccae (D. baccae), D. bohemiae, Diaporthe celeris (D. celeris)* (Guarnaccia et al., 2018), *Diaporthe chamaeropis (D. chamaeropis)* (Lawrence et al., 2015), *Diaporthe. Cynaroidis* (Lesuthu et al., 2019) *Diaporthe cytosporella (D. cytosporella), Diaporthe eres (D. eres), D. foeniculina, Diaporthe helianthi (D. helianthi)* (Dissanayake et al., 2017; Guarnaccia et al., 2018; Farr and Rossman, 2019), *Diaporthe hispaniae (D. hispaniae), D. hongkongensis* (Dissanayake et al., 2017), *Diaporthe hungariae (D. hungariae)* (Guarnaccia et al., 2018), *D. kyushuensis* (Kajitani and Kanematsu, 2000), *D. nebulae* (Lesuthu et al., 2019) *Diaporthe neotheicola (D. neotheicola)* (Úrbez-Torres et al., 2013), *Diaporthe nobilis (D. nobilis)* (Dissanayake et al., 2017), *D. novem* (Lawrence et al., 2015), *D. perjuncta* (Mostert et al., 2001), *Diaporthe perniciosa (D. perniciosa)* (Stoykow and Denchev, 2006), *D. phaseolorum* (Dissanayake et al., 2017), *Diaporthe rudis (D. rudis)* (Guarnaccia et al., 2018), *Diaporthe serafiniae (D. serafiniae)* (Lesuthu et al., 2019), and *D. sojae* (Dissanayake et al., 2017). Among these species *D. ampelina* is the mostly reported species with 42 records in 12 countries. The present study introduces the three novel taxa *D. guangxiensis, D. hubeiensis*, and *D. viniferae* and three new host records: *D. gulyae, D. pescicola, and D. unshiuensis*.

*Diaporthe eres* was identified as the most prominent and widespread species associated with grapevine dieback in China (37.5% of total isolates). Other than on grapevines, *D. eres* has been reported on *Aralia elata (A. elata)* (Wu et al., 2012), *Camellia* species (Gao et al., 2016), *Citrus* species (Huang et al., 2015), peach (Dissanayake et al., 2017), and pear (Bai et al., 2015) plants in China, causing diebacks. *Diaporthe eres* has been reported in many countries, such as the USA (Úrbez-Torres et al., 2013; Lawrence et al., 2015), Croatia (Kaliterna et al., 2012), Greece (Thomidis and Michailides, 2009), Italy (Cinelli et al., 2016), Latvia (Lombard et al., 2014), Poland (Kowalski et al., 2016), Russia, Serbia (Petrovic et al., 2015), and South Africa (Van Niekerk et al., 2005; Lesuthu et al., 2019) causing diseases on grapevines. These reports reveal that *D. eres* has a diverse host range and a broad geographical distribution. The second most abundant taxon, *D. sojae*, has a wide range of hosts as well, including *Camptotheca acuminata (C. acuminata)* (Chang et al., 2005), *Glycine max, Cucumis melo* (Lehman, 1923; Santos et al., 2011), *Capsicum annuum (C. annuum)* (Pennycook, 1989), *Stokesia laevis (S. laevis)* (Sogonov et al., 2008), and *Helianthus annuus (H. annuus)* (Thompson et al., 2011). These two *Diaporthe* species were previously identified and characterized from grapevines in China by Dissanayake et al. (2015a).

The present study recorded three *Diaporthe* species, *D. gulyae, D. pescicola, and D. unshiuensis*, associated with *Vitis* dieback for the first time. *Diaporthe gulyae* was previously reported on *H. annuus* in Australia (Thompson et al., 2011), Canada, and the United States (Mathew et al., 2015a,b) and on *Carthamus lanatus (C. lanatus)* in Italy (Andolfi et al., 2015). *Diaporthe pescicola* was previously described in association with peach shoot dieback in China (Dissanayake et al., 2017). *Diaporthe unshiuensis* was first described in China in 2015 as an endophyte of a *Citrus* sp. (Huang et al., 2015).

The identification and characterization of novel taxa and new host records is an indication of the high potential of *Diaporthe* to evolve rapidly. Host switching is often related to fungal adaptive ability (Bleuven and Landry, 2016). The changing environments and human interference present both challenges and opportunities for fungi, with some capable of switching from endophytic or saprobic lifestyles to pathogenic styles or becoming more aggressive and colonizing new hosts (Manawasinghe et al., 2018). The novel taxa and the new records reported here for grapevine trunk diseases in China might be due to these factors. During the past decade, northern China has become significantly warmer (Piao et al., 2010). The increased temperature could attract new pests and disease agents to the region. On the other hand, human-mediated factors can also influence the development of a new disease (McDonals, 2004). For example, in commercial grape vineyards, significant amounts of chemicals are applied annually in the form of pesticides and fungicides (Úrbez-Torres, 2011). Such applications could lead to the development of resistant strains of the target organism and non-target micro-fungi (Manawasinghe et al., 2018). Over time, strains and species that are more resistant and/or more aggressive could emerge. The recent identification of new species and new host records of *Diaporthe* in China and in Europe are consistent with the hypothesis. Studying the genetic diversity of pathogens provides clues to how host switches might have occurred and the genetic basis for new pathogen emergence.

The knowledge of the genetic diversity of a particular phytopathogen can be used to develop sustainable management strategies such as resistance breeding and fungicide screening. In this study, *D. eres* was analyzed, as it had a relatively large number of isolates from which to obtain reasonable estimates of various intraspecific diversity indices. In this study, multi-locus sequences were used as the marker of choice. The use of sequence data as genetic markers facilitated the analysis of genetic variations among isolates within a population. We selected ITS, β-tubulin, HIS, EF-1α, and CAL gene regions, as they were extensively used in phylogenetic analysis of the genus *Diaporthe*. In addition, ACT and Apn2 genes were selected since those regions provide a large number of polymorphic sites for the *Diaporthe eres* species complex (Udayanga et al., 2014b). Genetic polymorphisms are required for both phylogenetic and population genetic studies (Xu, 2006). Using these gene regions, we calculated haplotype richness (h_R_), the total number of haplotypes, Watterson's theta (Θ_w_), and pairwise nucleotide diversity (JI) for *Diaporthe eres* obtained from Chinese vineyards.

The combined effect of the mutation, recombination, marker ascertainment, and demography of a particular species can be revealed by analyzing and comparing gene genealogies and haplotype diversities within and between genes (Stumpf, 2004; Xu, 2006). The calculated haplotype diversities of *Diaporthe eres* were higher than 0.5 for Apn2, CAL, HIS, β-tubulin and the combined data, reflecting high genetic diversity. Tajima's D indicates how much population variation can be sustained over time (Tajima, 1989). In the present study, positive D values were observed for coding gene regions (Apn2, CAL, and HIS). This might be due to selective pressure causing a recent population contraction. The selection pressure could have come from the continuous application of fungicides, leading to the loss of certain genotypes. In contrast, Tajima's D for the combined sequences was negative (−0.20416), which indicates a possible recent population expansion of certain multi-locus genotypes (Tajima, 1989). In Hubei, several multi-locus genotypes were over-represented, consistent with this hypothesis.

The Hudson and Kaplan (1985) index for the recombination between Chinese and European isolates was calculated for this study. In our analysis, we calculated the number of recombination events in the history of a sample of sequences (R) and the number of recombination events that can be parsimoniously inferred from a sample of sequences (Rm) (Hudson, 1983; Kelly, 1997). When the rate of recombination equals zero, R gives zero (Hudson, 1983; Hudson and Kaplan, 1985). Since the R is given a value based on the history of the sample, Rm denotes the minimum number of recombination events implied by the data using the four-gamete test. A positive ZZ value, which reflects intragenic recombination, has played an important role in nucleotide variation and a high number of recombination events (Hudson, 1983). Therefore, we can conclude that recent recombination events might have occurred between the Chinese and European isolates. Haplotype networks provide a better understanding of the coexistence of ancestral and derived haplotypes by providing an account for recombination (Huson and Bryant, 2006). Therefore, haplotype networks are intensively used in intraspecific analyses. We used a median-joining network in which the number of mutations separate haplotypes (Castelloe and Templeton, 1994). In each network, the ancestral haplotype was predicted based on rooting probability (Posada and Crandall, 2001). The analyses suggested that the most recent ancestry of the Chinese haplotypes was shared with the Spanish and Hungarian haplotypes. In addition, haplotypes from the UK and Czech Republic shared ancestry with Chinese haplotypes. Overall, the *Diaporthe* population in China is genetically diverse and might have an admixture population. The current population is likely derived from a combination of endemic *D. eres* strains and introduced strains from other regions.

## Conclusion

Present study provides an account of *Diaporthe* species associated with Chinese vineyards by their phylogenetic placements. Collectively, in the present study, 111 *Diaporthe* strains were isolated and characterized into eight species using both morphological and molecular phylogenetic approaches. To identify those taxa, four gene regions were examined. The combination of ITS, CAL, β-tubulin, and EF-1α genes gave the best species delimitation in the genus *Diaporthe*. The present study introduced three novel taxa and three host records of *Diaporthe* associated with Chinese grapevines. The most abundant *Diaporthe* species was *D. eres*, which was moderately aggressive. *D. gulyae* was the most aggressive among the eight species on detached green shoots. The Chinese *D. eres* population was high in nucleotide diversity and haplotype diversity. In haplotype network analysis, the Chinese population was dispersed in the network but showed a certain degree of clustering according to their geographical origins. This result suggests that there is likely geographic structuring of *D. eres* in China. However, more in-depth analysis is required using more isolates from different provinces. Haplotype networks including Chinese and European isolates suggest a close relationship between the two populations. This is confirmed by the recombination among isolates from these two regions. Our results suggest that the *D. eres* population in China might be a result of an admixture. The results presented here provide opportunities for several fields, including grapevine breeding for disease-resistant cultivars, screening for new fungicides, and developing appropriate quarantine and management strategies to prevent and control grapevine dieback diseases.

## Data Availability

The sequence data generated in this study is deposited in NCBI GenBank (https://www.ncbi.nlm.nih.gov/genbank) and the respective accession numbers are given in Table 2. The Alignment generated in the present study available in TreeBASE (https://treebase.org/treebase-web/home.html) under the 24324.

## Author Contributions

JY and XL conceived the research. JY, IM, AD, XL, and WZ planned the basic research. ML, YZ, and WSZ provided materials. IM and AD conducted the experiments and prepared manuscript. IM, AD, DW, and JX analyzed data. KH, SB, and JY revised the manuscript. All authors read and approved the final manuscript.

### Conflict of Interest Statement

The authors declare that the research was conducted in the absence of any commercial or financial relationships that could be construed as a potential conflict of interest.
